# Cardioprotective mechanism of SGLT2 inhibitor against myocardial infarction is through reduction of autosis

**DOI:** 10.1007/s13238-020-00809-4

**Published:** 2021-01-08

**Authors:** Kai Jiang, Yue Xu, Dandan Wang, Feng Chen, Zizhuo Tu, Jie Qian, Sheng Xu, Yixiang Xu, John Hwa, Jian Li, Hongcai Shang, Yaozu Xiang

**Affiliations:** 1 Shanghai East Hospital, School of Life Sciences and Technology, Tongji University, Shanghai, 200092 China; 2 State Key Laboratory of Bioreactor Engineering, Shanghai Key Laboratory of New Drug Design, East China University of Science and Technology, Shanghai, 200237 China; 3 Section of Cardiovascular Medicine, Department of Internal Medicine, Yale Cardiovascular Research Center, Yale University School of Medicine, New Haven, CT 06511 USA; 4 Key Laboratory of Chinese Internal Medicine of Ministry of Education and Beijing, Dongzhimen Hospital Affiliated to Beijing University of Chinese Medicine, Beijing, 100700 China

**Keywords:** myocardial infarction, SGLT2 inhibitors, empagliflozin, cardioprotection, NHE1, autosis

## Abstract

Sodium-glucose cotransporter 2 (SGLT2) inhibitors reduce cardiovascular mortality in patients with diabetes mellitus but the protective mechanism remains elusive. Here we demonstrated that the SGLT2 inhibitor, Empagliflozin (EMPA), suppresses cardiomyocytes autosis (autophagic cell death) to confer cardioprotective effects. Using myocardial infarction (MI) mouse models with and without diabetes mellitus, EMPA treatment significantly reduced infarct size, and myocardial fibrosis, thereby leading to improved cardiac function and survival. In the context of ischemia and nutritional glucose deprivation where autosis is already highly stimulated, EMPA directly inhibits the activity of the Na^+^/H^+^ exchanger 1 (NHE1) in the cardiomyocytes to regulate excessive autophagy. Knockdown of NHE1 significantly rescued glucose deprivation-induced autosis. In contrast, overexpression of NHE1 aggravated the cardiomyocytes death in response to starvation, which was effectively rescued by EMPA treatment. Furthermore, *in vitro* and *in vivo* analysis of NHE1 and Beclin 1 knockout mice validated that EMPA’s cardioprotective effects are at least in part through downregulation of autophagic flux. These findings provide new insights for drug development, specifically targeting NHE1 and autosis for ventricular remodeling and heart failure after MI in both diabetic and non-diabetic patients.

**Electronic supplementary material:**

The online version of this article (10.1007/s13238-020-00809-4) contains supplementary material, which is available to authorized users

## INTRODUCTION

Diabetes mellitus (DM) is a rapidly growing major global health problem, portending an increased risk of cardiovascular events, heart failure and death (Greene et al., 2018; Wang et al., 2018; Zelniker and Braunwald, 2018). Although hyperglycemia is a strong risk factor for microvascular complications associated with DM, the consequences of intensive glycemic control on macrovascular complications remain unproven. It took more than 10 years for prospective studies to demonstrate a significant reduction in myocardial infarction and death with metformin (Holman et al., 2008). In fact, various glucose-lowering agents increase the risk of hospitalization for heart failure (Nassif and Kosiborod, 2018). Initial concerns about the cardiovascular safety of Rosiglitazone (Nissen and Wolski, 2007) led the U.S. Food and Drug Administration (FDA) in 2008 (followed by the European Medicines Agency) to mandate that new glucose-lowering agents be tested for cardiovascular safety post-marketing. During the last decade, double-blind, placebo-controlled trials for Sitagliptin and Saxagliptin several were conducted, with neutral effects on cardiovascular outcomes (Scirica et al., 2013; Green et al., 2015). These were followed by the identification of unanticipated cardiovascular benefits with some newer generation drugs, including the sodium-glucose co-transporter-2 (SGLT2) inhibitors.

Empagliflozin (EMPA), an SGLT2 inhibitor, approved by the FDA in 2014, significantly reduced cardiovascular mortality and heart failure hospitalization (Zinman et al., 2015). Since then, other SGLT2 inhibitors, Canagliflozin (Neal et al., 2017) and Dapagliflozin (Wiviott et al., 2019), have been found to have cardiovascular protection in patients with type 2 diabetes mellitus (Zelniker et al., 2019). As such, the 2018 updated US and European treatment guidelines for diabetes mellitus incorporated SGLT2 inhibitors as second line glucose lowering agents after metformin. A very recent clinical trial (DAPA-HF Trial) demonstrated that in patients with heart failure and reduced ejection fraction, the risk of worsening heart failure or death from cardiovascular causes was lower in those who received SGLT2 inhibitor dapagliflozin than in those who received placebo, regardless of the presence or absence of diabetes (McMurray et al., 2019; Nassif et al., 2019). Whether the effect of SGLT2 inhibition on outcomes in non-diabetic heart failure patients is class effect or drug-specific effect remains unclear (Packer et al., 2017; Maack et al., 2018; Santos-Gallego et al., 2019; Yurista et al., 2019). In addition, although well tolerated, there are known adverse effects with SGLT2 inhibitors that require clinical monitoring, such as genital mycotic infections, diabetic ketoacidosis, volume depletion particularly in the setting of concomitant diuretic use, and lower limb amputations (with canagliflozin) (Taylor et al., 2015; Ueda et al., 2018; Zheng et al., 2018; Perry et al., 2019).

SGLT2 inhibitors mainly act on the kidney SGLT2, excreting excess glucose. However, they also significantly reduces cardiovascular mortality and heart failure admission rate, through an unknown mechanism independent of glucose (Bell and Yellon, 2018; Nassif and Kosiborod, 2018). Given that cardiomyocytes do not express SGLT2, whether the drug acts directly on the heart to produce cardioprotection and related direct protective molecular mechanisms need to be elucidated.

Na^+^/H^+^ exchanger 1 (NHE1) is mainly expressed in cardiomyocytes, and its activity is significantly increased under the pathological conditions of diabetes, heart failure and acute ischemia-reperfusion injury (Packer, 2017). Activation of NHE1 increases cardiomyocyte intracellular sodium load, resulting in calcium overload during ischemia-reperfusion and aggravates reperfusion injury. Consistently, NHE1 knockout mice exhibited myocardial ischemia-reperfusion injury tolerance (Wang et al., 2003). In contrast, cardiomyocyte-specific overexpression of NHE1 induced cardiac hypertrophy and heart failure in mice (Nakamura et al., 2008). Recent proposed hypothesis for the mechanism of cardioprotection of SGLT2 inhibitors suggest it may be through induction of autophagy (Avogaro et al., 2020; Packer, 2020a, b). We set out to establish the protective effects of EMPA during MI.

At the initiation of our studies, we sought to use pharmacological, genetic and unbiast screening approaches to determine the molecular mechanisms for SGLT2 inhibitor, EMPA, on improved cardiac function and remodeling after MI. Using genetic mouse models, we demonstrated that SGLT2 inhibitor improved cardiac function and survival post MI. Based on a screen of membrane transporter/ion channel compound library, molecular docking prediction and pharmacological testing of drug targets, we demonstrated that NHE1 is the main target of SGLT2 inhibitors on cardiomyocytes. We further demonstrated that EMPA’s cardioprotective effects are through NHE1 mediated downregulation of excessive autophagic flux. Autophagy is a complex evolutionarily conserved intracellular process in response to a variety of stimuli, including cellular stress, ischemic injury and nutritional starvation. Both insufficient activation of autophagy and excessive autophagy may be harmful, particularly in the setting of myocardial infarction (Liu et al., 2018; Santulli, 2018; Sciarretta et al., 2018c). EMPA appears to regulate and optimize this important autophagy mechanism in the heart.

## RESULTS

### Effects of empagliflozin on diabetic and non-diabetic mice with myocardial infarction

**Figure 1:**
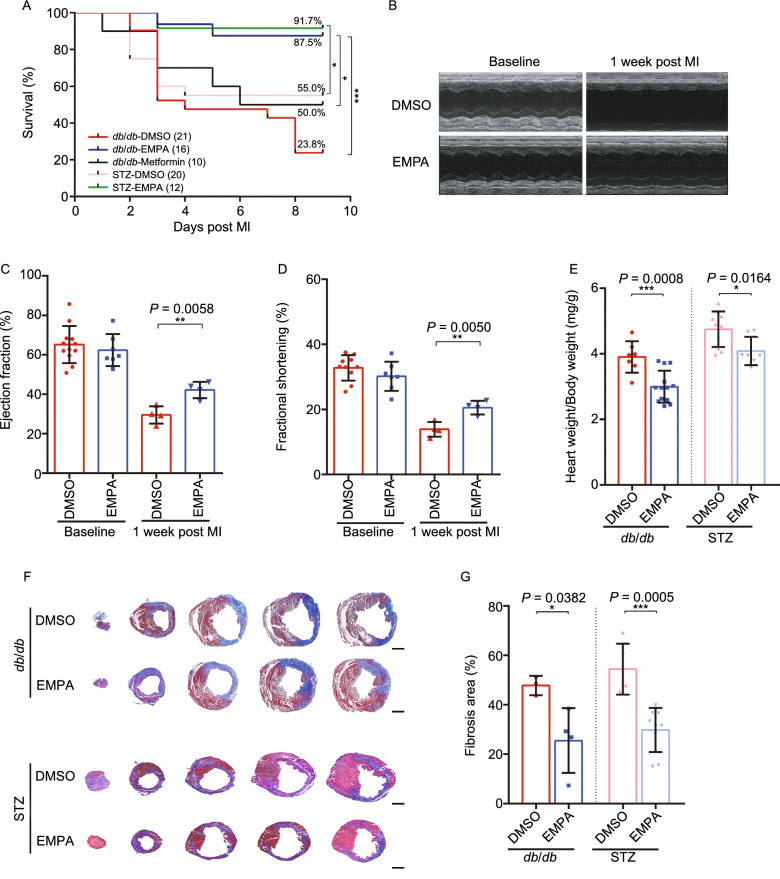

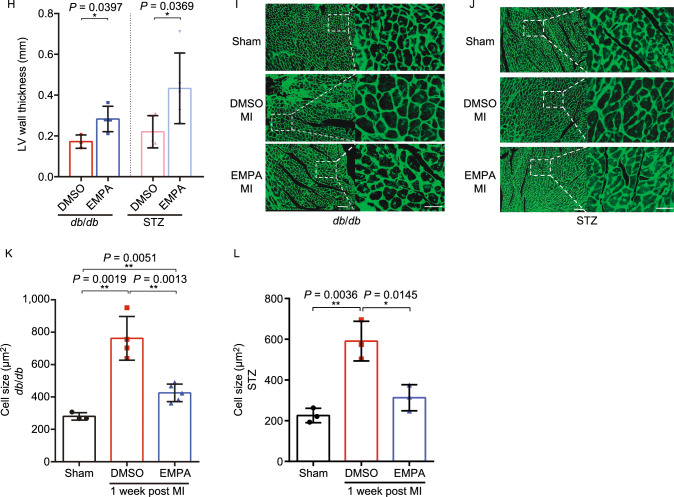
**Empagliflozin attenuates acute myocardial ischemia injury in diabetic mice**. (A) Survival curve (log-rank [Mantel-Cox] test) of *db*/*db* mice and STZ-induced diabetic mice (with or without EMPA or metformin therapy) and STZ-induced diabetic mice (with or without EMPA therapy) subjected to ligation of left anterior descending coronary artery (LAD) followed by observation for 1 week. (B) Representative M-mode echocardiographic tracings from *db*/*db* mice (with or without EMPA therapy). (C and D) Cardiac function was evaluated from M-mode images means ± SD. (E) A comparison of EMPA and control on heart/body weight. (F–H) Representative photographs and quantitative data of Masson’s trichrome staining and left ventricular wall thickness of heart sections. (I–L) Wheat germ agglutinin (WGA) staining for cardiomyocyte cell size in *db*/*db* and STZ-induced diabetic mice exposed to EMPA. Scale bars, 100 μm. All data are presented as mean ± SD, *P < 0.05, **P < 0.01, ***P < 0.001

Prompted by recent reports that EMPA promotes reverse cardiac remodeling in patients with type 2 diabetes (T2DM), we sought to determine the effect of SGLT2 inhibitor EMPA on infarcted diabetic murine hearts. To this end, we systematically performed sham or left anterior descending (LAD) coronary artery ligation on a total of 56 *db*/*db* mice (T2DM model). In addition to a sham group, the LAD-operated mice were randomized to groups pretreated with either DMSO (vehicle control), EMPA, Metformin or post-treatment EMPA (Fig. S1A and S1C). At one week after surgery, administration of EMPA before LAD surgery significantly improved *db*/*db* mice survival compared to DMSO (87.5% vs. 23.8%, *P* = 0.0002) or Metformin treatment (87.5% vs. 50%, *P* = 0.0334) (Fig. 1A). Survival in the MI-Metformin and the MI-DMSO (50% vs. 23.8%) were not statistically significant (Fig. 1A). More importantly, although there was a marked reduction in left ventricular systolic function following MI in both groups (MI-DMSO and MI-EMPA), significant improvement of systolic function was observed only in the EMPA group. (Fig. 1B–D and Table S1). Consistently, heart weight to body weight ratio in the EMPA-treated mice was significantly decreased compared to that of the mice in the other treatment groups (Fig. 1E). We further determined the effect of EMPA treatment on cardiac structural remodeling and functional performance. Histological analysis and quantification of the scar showed that the EMPA-treated hearts had significant reduction in fibrotic scar size (Fig. 1F and 1G) and increased LV wall thickness (Fig. 1H). In addition, EMPA-treated hearts demonstrated a significant decrease in cardiomyocyte size in the MI border zone, but not the remote zone, indicative of an attenuated hypertrophic response Fig. 1I and 1K).

We then examined whether EMPA alleviated cardiac dysfunction in LAD ligation-operated Streptozotocin (STZ)-induced diabetic mice (mimic type 1 diabetes, *n* = 43) (Fig. S1B and S1D). Consistent with what we have observed with *db*/*db* diabetic model, administration of EMPA resulted in the improvement of survival 1 week after MI compared with MI-DMSO groups (91.7% vs. 55.0%, *P* = 0.0325) (Fig. 1A) decreased heart weight to body weight ratio (Fig. 1E), reduced fibrotic scars (Fig. 1F and 1G), and increased LV wall thickness (Fig. 1H). In addition, in EMPA-treated hearts, there was a significant decrease in cardiomyocyte size in the MI border zone, but not the remote zone (Fig. 1J and 1L). These studies further reinforce the beneficial effects of EMPA in multiple DM models.

**Figure 2:**
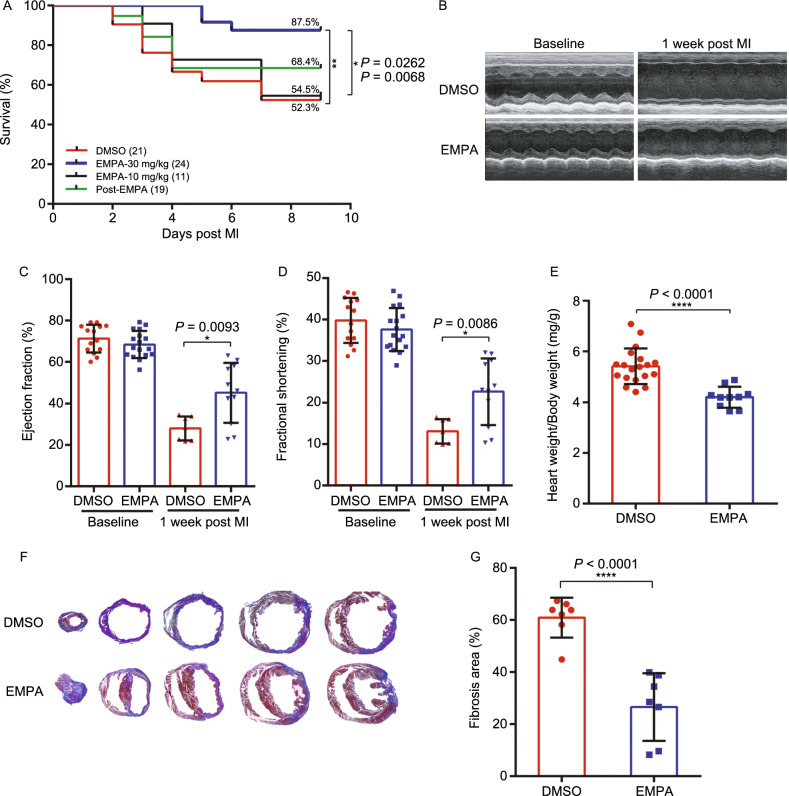

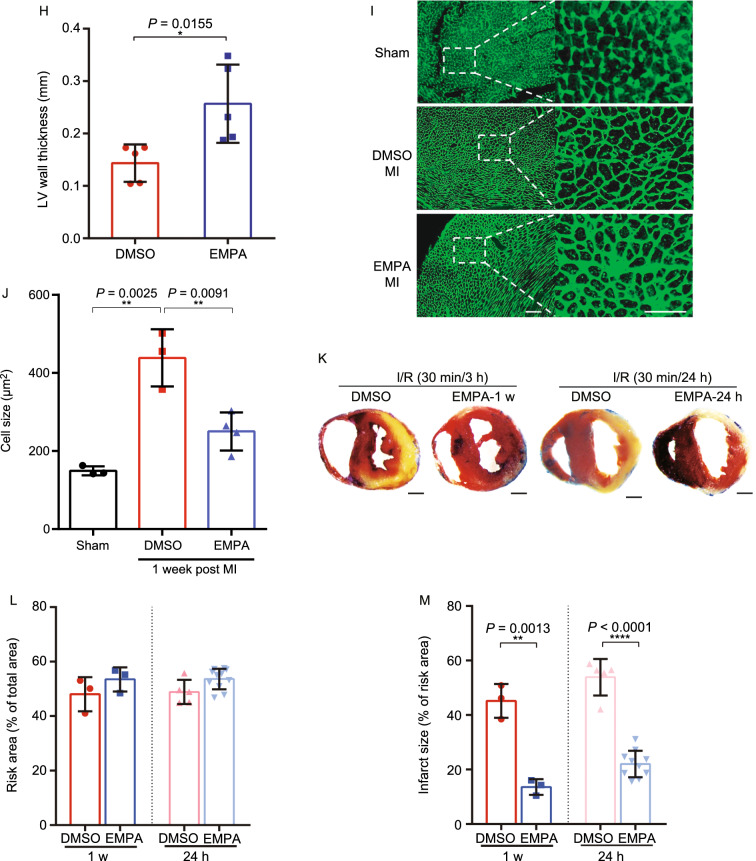
**Empagliflozin attenuates acute myocardial ischemia injury in WT mice**. (A) Survival curve (log-rank [Mantel-Cox] test) of WT (with or without EMPA therapy) subjected to ligation of left anterior descending coronary artery (LAD) followed by observation for 1 week. (B) Representative M-mode echocardiographic tracings from WT mice (with or without EMPA therapy). (C and D) Cardiac function was evaluated from M-mode images means ± SD. (E) A comparison of EMPA and control on heart/body weight. (F–H) Representative photographs and quantitative data of Masson’s trichrome staining and left ventricular wall thickness of heart sections. (I and J) WGA staining for cardiomyocyte cell size in WT mice. Scale bars, 100 μm. (K–M) Myocardial infarction size in response to ischemia/reperfusion (I/R) in WT mice treated with EMPA 1 week and 24 h prior to infarction. Representative photographs (upon) and quantitative data (down) for infarct size and risk area in hearts from DMSO and EMPA-treated mice subjected to I/R injury. Scale bar, 1 mm. EMPA-1W: pretreatment with EMPA for 1 week, 30 mg/kg/day; EMPA-24 h: pretreatment with EMPA four times for 24 h, 30 mg/kg/once. All data are presented as mean ± SD, *P < 0.05, **P < 0.01, ***P < 0.001, ****P < 0.0001

As SGLT2 inhibitors confer clinical cardiovascular benefits in heart failure patients with or without diabetes (McMurray et al., 2019; Nassif et al., 2019), we next examined whether EMPA reduced cardiac damage in LAD ligation-operated WT mice (*n* = 75 WT 10–12 week mice) (Fig. S2A). Administration of EMPA also improved the survival of LAD-ligation-operated WT mice (Fig. 2A), and suppressed myocardial infarction-induced adverse cardiac remodeling and dysfunction (Fig. 2B–J and Table S2).

Apart from utilizing LAD ligation-operated WT mice, we also compared the myocardial infarction size in response to ischemia/reperfusion (I/R) in WT mice treated with EMPA 1 week and 24 h prior to infarction (Figs. 2K and S2D). Remarkably, we observed that administration of EMPA to WT mice (1 week or 24 h prior to I/R), resulted in a significant reduction in infarct size as % of risk area (Fig. 2L and 2M). This observation further supports a myocardial protective role for EMPA for both diabetic and non-diabetic mice.

### Empagliflozin protects cardiomyocytes from glucose deprivation and binds to cell surface proteins

**Figure 3:**
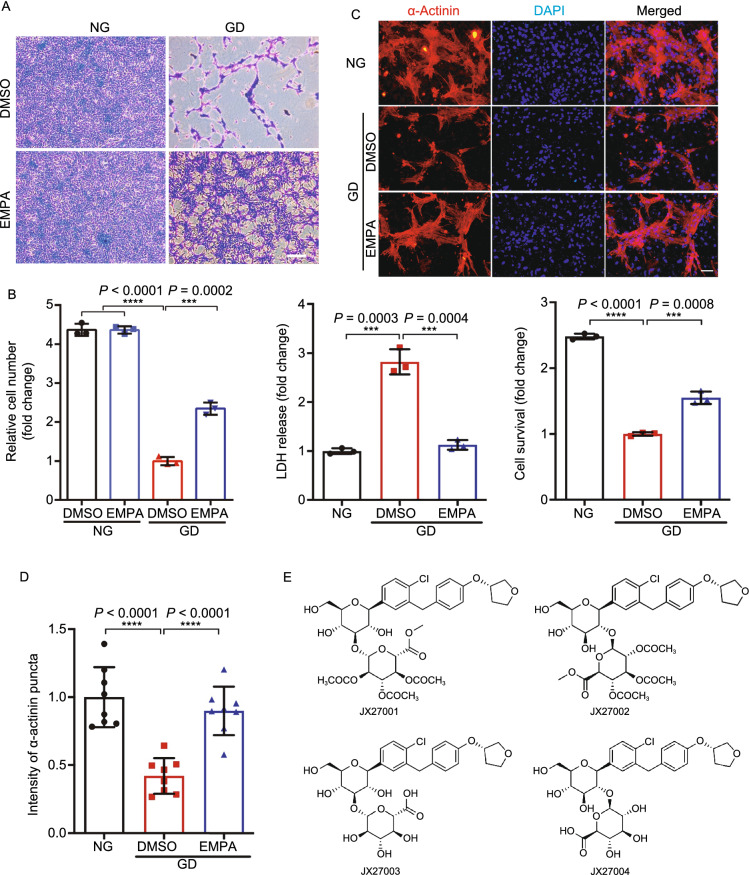

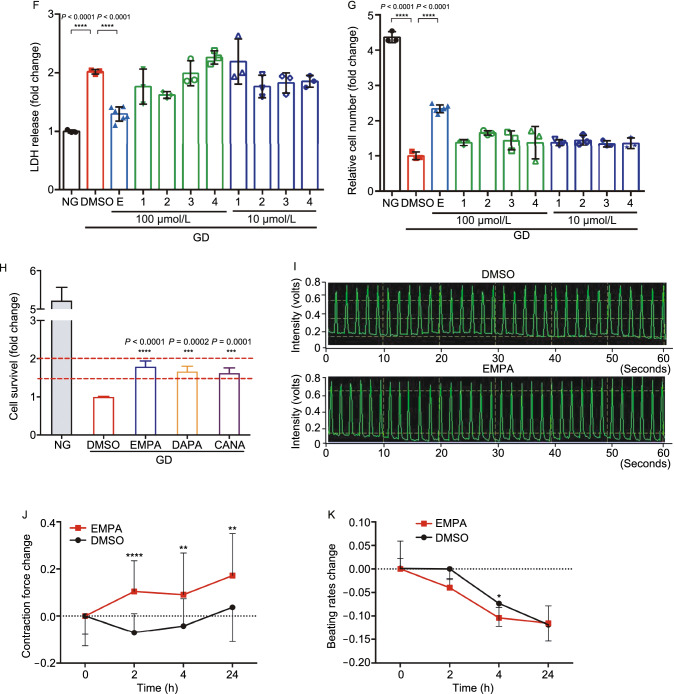
**Direct effects of empagliflozin on cardiomyocytes**. (A) Representative crystal violet staining of the H9c2 cell line exposed to GD for 24 h with or without EMPA treatment. (B) Quantification of H9c2 cell number, cell survival using CCK8 and LDH assay. All cell-number quantification experiments were performed in triplicate over three separate experiments. (C) Representative images of isolated neonatal rat cardiomyocytes exposed to GD with or without EMPA treatment. (D) Quantitative ratio of α-actinin positively labeled cardiomyocytes. (E) Chemical structures, known *in vivo* metabolites of EMPA in humans. (F–H) Quantification cell number and survival of H9c2 cell exposed to GD for 24 h with major metabolites (1–4), dapagliflozin (DAPA) and canagliflozin (CANA). (I–K) Representative images showing the contraction force curve measured by FelixGX detection system in neonatal rat cardiomyocytes with or without EMPA treatment. All data are presented as mean ± SD, *P < 0.05, **P < 0.01, ***P < 0.001, ****P < 0.0001

With the benefits being observed within a short time window (24–48 h pretreatment), the protective effects of EMPA is unlikely through atherosclerosis reduction. To investigate whether EMPA directly interacts with cardiomyocytes to confer its beneficial effects against myocardial injury, we established an *in vitro* cell culture model to mimic myocardial ischemia and/or reperfusion stress. When cultured cardiomyocytes exposed to glucose deprivation (GD) for 24 h, 70%–80% cells succumbed to death (Figs. 3A and S3A). Remarkably, pretreatment with EMPA for 48 h significantly rescued the GD-induced cell death (Figs. 3B and S3B). Moreover, EMPA significantly inhibited the GD-induced cardiomyocytes apoptosis (Fig. S10A and S10B). In addition, the protective effects of EMPA were also observed in neonatal rat cardiomyocytes subjected to glucose deprivation (Fig. 3C and 3D). By contrast, EMPA exhibited no effect on proliferation and migration of endothelial cells (Fig. S4). We further demonstrated that it was EMPA itself rather than its metabolites (Fig. 3E), that conferred the protective effects on cardiomyocytes against starvation (Figs. 3F, 3G and S3C). Interestingly, in the GD-induced cell model, other SGLT2 inhibitors (including DAPA and CANA) also exhibited cardioprotective effects, indicative of common beneficial effects conferred by the entire class of SGLT2 inhibitors (Fig. 3H). In primary isolated cardiomyocytes, we further observed that EMPA improved myocyte contractility without affecting their beating frequency (Figs. 3I–K, S3F and S3G).

**Figure 4:**
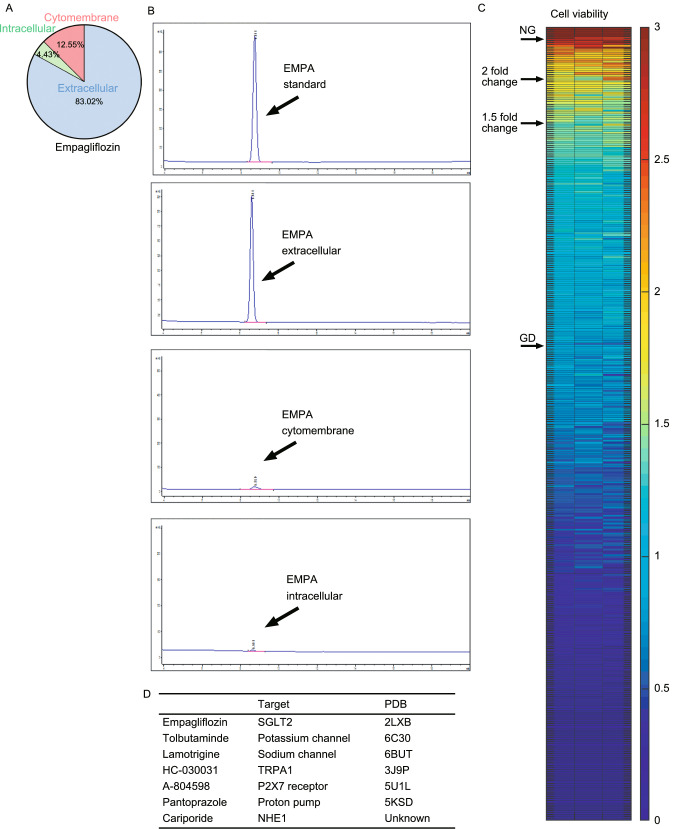

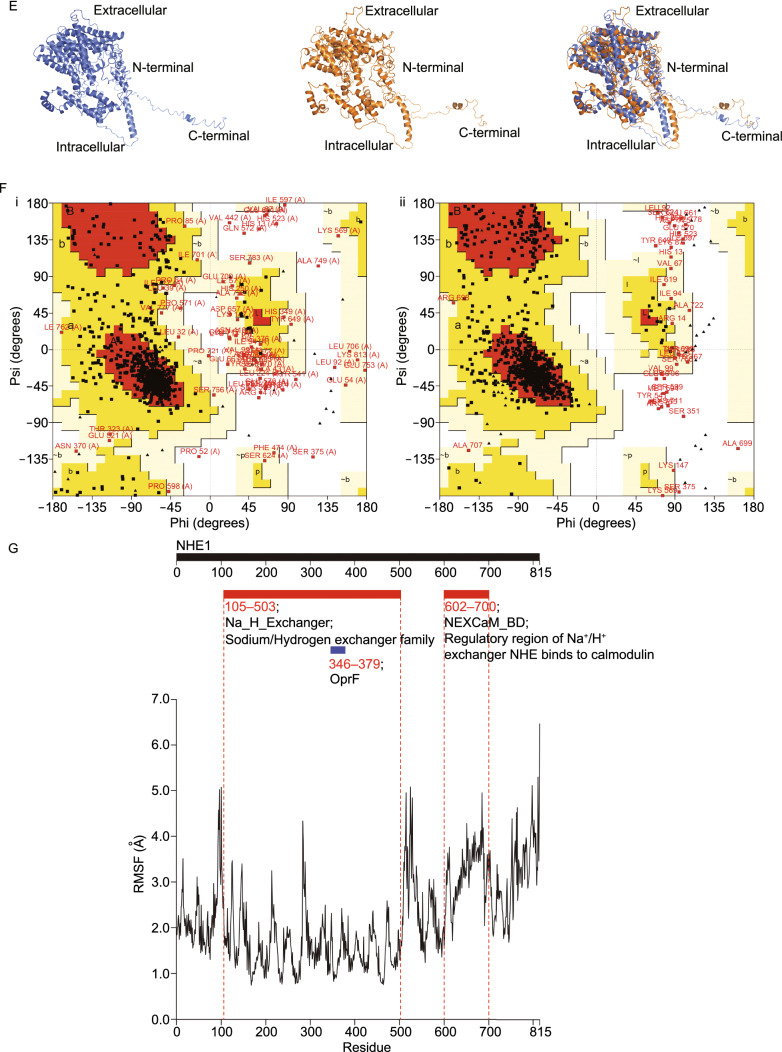
**A screen of membrane proteins and in silico analysis of SGLT2 inhibitors binding**. (A) Diagrammatic representation of the EMPA proportion of extracellular fluid, intracellular fluid and cell membrane on neonatal rat cardiomyocytes that were treated with EMPA-48 h. (B) HPLC analysis of the Empagliflozin from the neonatal rat cardiomyocytes of extracellular fluid, intracellular fluid and cytomembrane. (C) A screen of membrane transporter/ion channel compound library in GD-induced cardiomyocytes stress model. (D) Representative compounds or inhibitors over 1.5-fold difference, and individual targets and structure PDB information. (E) The predicted 3D model of NHE1 from I-TASSER (i), the optimized model using GROMACS (ii) and their alignment (iii). (F) Ramachandran plot analysis of the NHE1 model from ITASSER (i) and the optimized model using GROMACS (ii). (G) The predicted promising functional motifs present in NHE1

Since cardiomyocytes do not express SGLT2, EMPA must act on other target(s) to exert its beneficial effect on cardiomyocytes. We thus extracted the cellular fractions (membrane, cytoplasm, nucleus, etc.) and supernatants from the EMPA treated cell samples and utilized HPLC to analyze the distribution of EMPA in cultured cardiomyocytes (tamoxifen as control) (Fig. S5). We found that a large number of EMPA was associated with serum proteins in the culture medium and remained in the extracellular fluid whereas the remainder mostly bound to the cell membrane (Fig. 4A and 4B). Therefore, EMPA most likely acts on another membrane transporter/ion channel protein to elicit the cardioprotective effects. Utilizing the in vitro cell culture models mentioned above, we performed an unbiased pharmacological screen targeting potential membrane transporter/ion channel. As shown in Fig. 4C and 4D, inhibitors targeting potassium channel, sodium channel, proton pump, NHE1, P2X7 receptor and transient receptor potential A1 exhibited a comparable effect on cardiomyocytes in response to GD stress. Therefore, these specific membrane transporters and ion channels are potential candidate targets of SGLT2 inhibitors including EMPA expressed in cardiomyocytes.

### Empagliflozin binds cardiomyocyte NHE1 *in silico*

**Figure 5:**
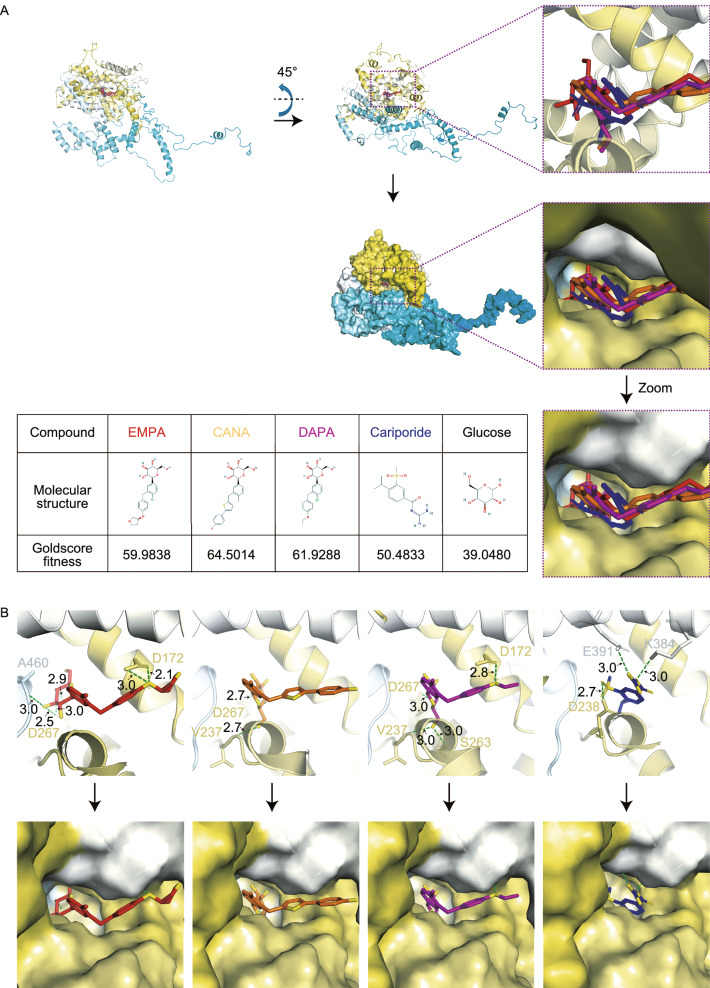
***In silico*** **analysis of SGLT2 inhibitors binding to a structural model of NHE1**. Comparison of binding affinities of three SGLT2 inhibitors CANA, DAPA and EMPA and one known NHE1 inhibitor Cariporide against NHE1 optimized structural model. Cariporide is shown in blue, CANA is shown in orange, DAPA in purple, and EMPA in red. Glucose is served as negative control

We then conducted *in silico* analysis of EMPA binding to the crystal structure (potassium channel [6C3O], sodium channel [6BUT], P2X7 [5U1L], and TRPA1 [3J9P]) or a structure model of NHE1 (Fig. 4D). The flowchart for alignment, homology modeling, fold recognition, evaluation and optimization of model, molecular docking and visualization was shown in Fig. S6. NHE1 appeared to be the most plausible candidate. Since homology below 30% sequence identity limits single-sequence based searches, the molecular modelling for human NHE1 was carried out by iterative threading ASSEmbly refinement (I-TASSER) server, an integrated platform for automated protein structure and function prediction based on the sequence-to-structure-to-function paradigm (Todd et al., 2001; Yang and Zhang, 2015). The predicted 3D model of NHE1 from I-TASSER (i), the optimized model using GROMACS (ii) and their alignment (iii) was shown in Fig. 4E. In addition, we applied ramachandran plot analysis of the NHE1 model from I-TASSER and the optimized model using GROMACS (Figs. 4F and S7). The predicted promising functional motifs present in NHE1 were depicted in Fig. 4G. As shown in Fig. 5A and 5B, all three SGLT2 inhibitors (Empagliflozin, Canagliflozin, Dapagliflozin) and one known NHE1 inhibitor Cariporide displayed high binding affinity to NHE1 with Goldscore fitness value of 59.98, 64.50, 61.93 and 50.48, respectively. Binding of a glucose molecule to the NHE1 structure model demonstrated that glucose bound in an identical orientation to the glucoside part of the SGLT2 inhibitors, but with a lower Goldscore fitness value of 39.05, suggesting the importance of the hydrophobic part of the SGLT2 inhibitors to ensure efficient binding (Fig. 5B). The binding pockets for the four SGLT2 inhibitors are shown in Fig. S8.

### Empagliflozin targets NHE1 to confer cardioprotective effects

**Figure 6:**
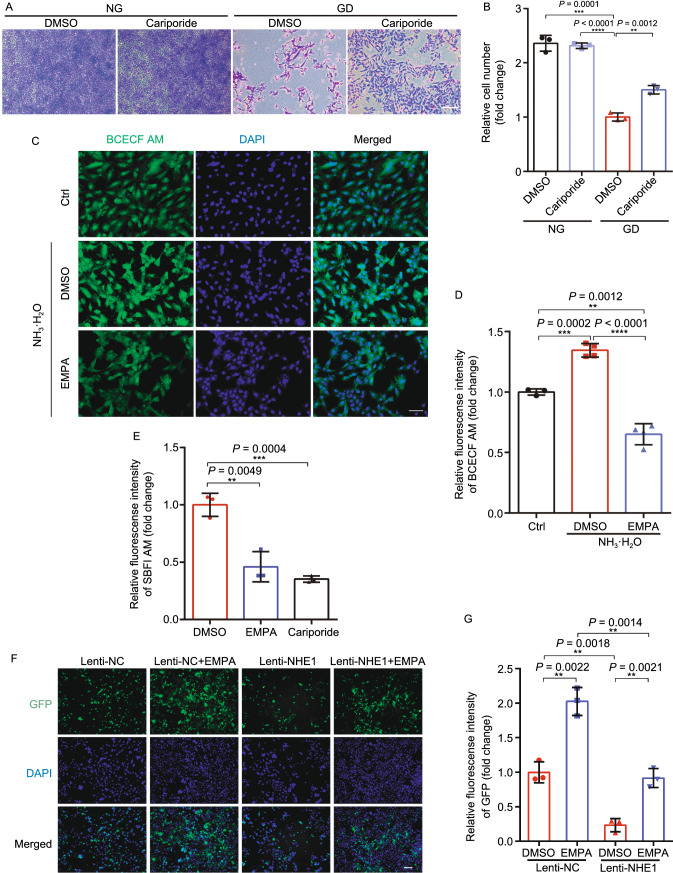

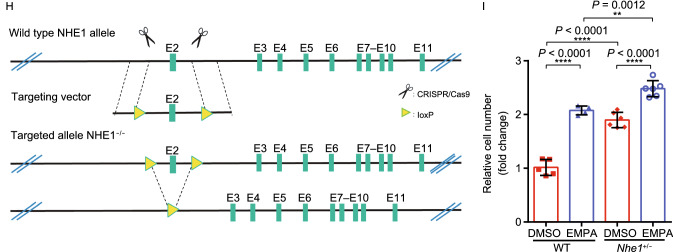
**Empagliflozin targeting NHE1 to confer cardioprotective effects**. (A) Representative crystal violet staining of the H9c2 cell line exposed to GD for 24 h with or without NHE1 inhibitor, cariporide, treatment. (B) Quantification of H9c2 cell number using crystal violet staining. All cell-number quantification experiments were performed in triplicate over three separate experiments. (C and D) Intracellular pH of H9c2 (with or without EMPA treatment) in NH_3_·H_2_O condition was detected by staining with BCECF-AM. Scale bar, 100 μm. (E) The Fluorescence fold change of H9c2 cells stained with SBFI meanwhile exposure to incubated with EMPA or cariporide 40 min assessing intracellular sodium ion concentration. SBFI, sodium-binding benzofuran isophthalate. (F and G) Representative images and quantification for GFP (Lenti-NC and Lenti-NHE1) on Lenti-transduced H9c2 treated with EMPA exposed to GD for 24 h. Scale bar, 100 μm. (H) The schematic diagram used CRISPR/Cas9 technology to edit the Nhe1 gene (BIOCYTOGEN). (I) Quantitation of cell numbers from cardiac specific *Nhe1*^+/−^ mice exposed to GD (with or without EMPA therapy) for 24 h. All data are presented as mean ± SD, *P < 0.05, **P < 0.01, ***P < 0.001, ****P < 0.0001

If the cardiac protective effect of EMPA is through inhibiting NHE1 activity, we would expect that previously identified NHE1 inhibitor could exert similar effect. Indeed, we found that the known NHE1 inhibitor cariporide protected cardiomyocytes from starvation-induced cell death (Fig. 6A and 6B). As activation of NHE1 increases cytoplasmic Na^+^ and Ca^2+^ concentrations and decreased mitochondrial Ca^2+^ concentration, thereby inducing cardiomyocyte death and eventually causing heart failure, we measured the cytoplasmic Na^+^ and Ca^2+^ concentration in EMPA-treated cardiomyocytes. We found that EMPA treatment reduced cytoplasmic Na^+^, pH and Ca^2+^ concentration **(**Figs. 6C–E and S9A–C**)**. Interestingly, overexpression of NHE1 rendered cardiomyocytes more sensitive to glucose deprivation while EMPA reversed the detrimental effects of overexpression of NHE1 on cardiomyocytes (Figs. 6F, 6G and S9G–K). To determine the functional significance of NHE1 inhibition on the protective effect of EMPA, we used isolated cardiomyocytes from cardiac specific NHE1 knockout mice (Fig. 6H) pretreated with EMPA and then subjected these cardiomyocytes to glucose deprivation. Although EMPA significantly attenuated glucose deprivation-induced cardiomyocytes death from WT mice, the beneficial effects of EMPA were blunted in isolated cardiomyocytes from cardiac specific NHE1 KO mice (Fig. 6I), which suggest that the effect of EMPA is through cardiac specific NHE1.

### Empagliflozin’s cardioprotective effects are through downregulation of autophagic flux and autosis

**Figure 7:**
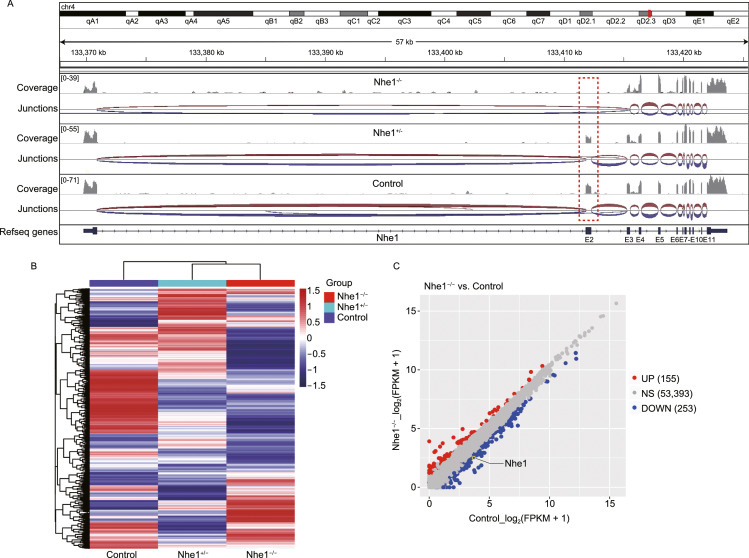

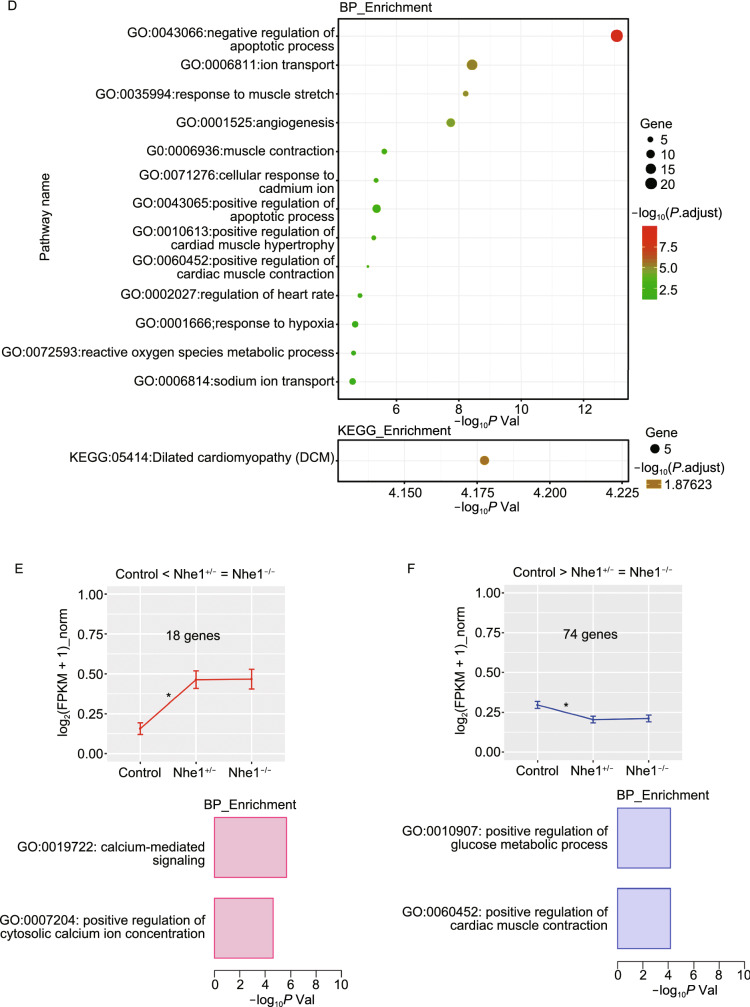

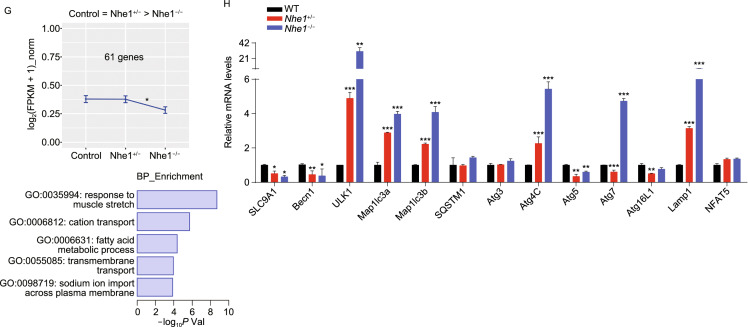
**Gene expression profile in NHE1 knockout mice heart**. RNA-seq data of 3 samples, a wildtype, a heterozygous (*Nhe1*^+/−^) (Hetero), as well as a homozygous (*Nhe1*^−/−^) mouse (Homo), were obtained using Illumina sequencing platform. (A) The aligned sequencing reads of NHE1 of such four samples on IGV were depicted. (B) A hierarchical clustering algorithm was used to group samples on the basis of similarity in the patterns with which their expression varied over these genes. (C–G) Identifying the expression patterns. GFOLD was used to call the differentially expressed genes (DEGs). (H) Analysis of gene expression in autophagy pathway with real-time PCR array for the neonatal mouse cardiomyocytes (WT, *Nhe1*^+/−^*, Nhe1*^−/−^). Gene expression was calculated as fold change relative to WT neonatal mouse cardiomyocytes. All values are mean ± SD (*n* = 3), *P < 0.05, **P < 0.01, ***P < 0.001

To determine the molecular mechanism underlying the cardioprotective effect of inhibiting NHE1 activity, we adopted a further unbiast screening approach and performed RNA-seq (transcriptome profiling). Three clusters of genes were identified highly associated with the knockout of NHE1 (Figs. 7, S12 and S13). Function enrichment of genes in such clusters identified ion transport, glucose metabolic process, and cell death pathways. Several specific genes, implicated in the induction of autophagy (Zhang and Ney, 2009), were significantly downregulated with knockout of NHE1 (Fig. 7H).

**Figure 8:**
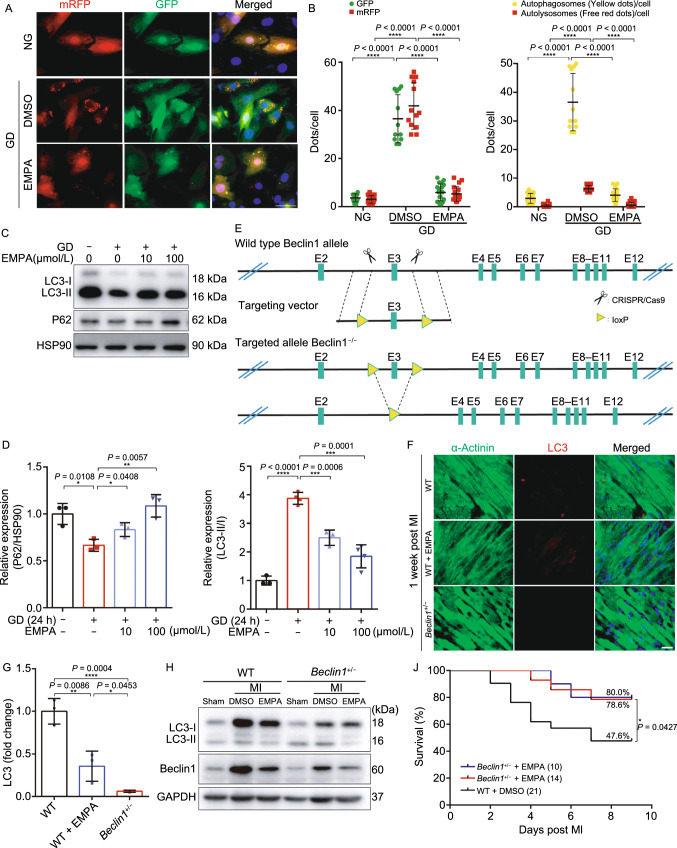

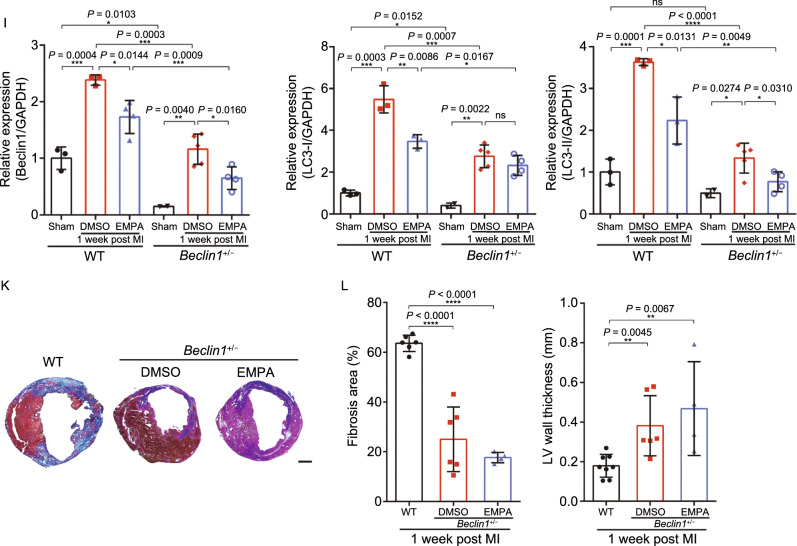
**Empagliflozin downregulated autophagy of cardiomyocytes**. (A and B) EMPA reduced the formation of autophagosome and autolysosome. Cardiomyocytes were infected with an adenovirus expressing mRFP-GFP tandem fluorescent-tagged LC3 (tfLC3). Scale bar, 50 μm. (C and D) Western blot analysis of the neonatal rat cardiomyocytes autophagy response to indicated concentrations of EMPA. (E) The schematic diagram used CRISPR/Cas9 to edit the beclin1 gene. (F) Representative immunofluorescence images showing LC3 staining in WT mice heart border tissue response to EMPA and *beclin1*^+/−^ post MI. (G) Quantification of the LC3 puncta number using ImageJ. (H) Western blot analysis of heart border tissue from sham, WT, WT treated with EMPA, *beclin1*^+/−^ and *beclin1*^+/−^ treated with EMPA post MI. Quantification was shown in (I). (J) Survival curve of WT and *beclin1*^+/−^ mice subjected to LAD followed by observation for 1 week. (K and L) Representative photographs and quantitative data of Masson’s trichrome staining and left ventricular wall thickness of heart sections. Scale bar, 1 mm. All data are presented as mean ± SD, *P < 0.05, **P < 0.01, ***P < 0.001, ****P < 0.0001

Glucose deprivation is known to induce autophagy (Matsui et al., 2007). Moreover, a recent study supports that during myocardial infarction ischemic/reperfusion injury, excessive autophagy leading to cardiomyocyte cell death (autosis) (Liu et al., 2013; Nah et al., 2020). As NHE1 knockout reduced autophagy-related genes, we tested whether EMPA may protect cardiomyocytes against starvation through inhibiting excessive autophagy and thus autosis. To analyze autophagic flux we assessed the effect of EMPA on the formation of both autophagosomes and autolysosomes by using an adenovirus expressing mRFP-GFP tandem fluorescent-tagged LC3 (tfLC3) (Hariharan et al., 2010). Monomeric RFP (mRFP), but not GFP, produces fluorescence in the acidic environment of lysosomes. Therefore, the colocalization of GFP and mRFP is indicative of autophagosomes, exhibiting yellow in the merged image. The free mRFP signal that does not overlay with the GFP in the merged image is indicative of autolysosomes (Kobayashi et al., 2012). GD enhanced active autophagic flux as evidenced by increased mRFP and GFP-LC3 individual signals and autophagosomes (yellow) (Figs. 8A and S10C). EMPA treatment dramatically reduced the number of mRFP and GFP-LC3 signals and autophagosomes (yellow) with GD, indicating that EMPA can block autophagic flux (Figs. 8B and S10D), consistent with the results from western blot analysis (Figs. 8C, 8D, S10E and S10F).

*In vitro* analysis may not reflect *in vivo*, moreover there has been recent hypothesis that the protective effects of EMPA may be through induction of autophagy (Avogaro et al., 2020; Packer, 2020b, a). Key to these *in vivo* studies were the earlier LAD ligation models (Figs. 1 and 2) which established the protective role of EMPA and the use of genetic suppression (not knockout) of autophagy using *beclin 1*^+/−^ mice (insensitive to autophagy inducers (Sciarretta et al., 2018c)) (Fig. 8E). Complete elimination of autophagy (knockout) as well as excess autophagy are both detrimental (Delbridge et al., 2017). Such an autophagy-targeted genetic approach is needed as EMPA may have other off target protective effects beyond autophagy. We thus analyzed heart tissue from WT and *beclin 1*^+/−^ mice subjected to LAD with immunostaining and western blot. Beclin 1 plays a central role in autosis (Nah et al., 2020). We demonstrated that the cleaved LC3 in WT mice heart 1 week post MI was significantly reduced when treated with EMPA, comparable with those in the *beclin 1*^+/−^ mice (Fig. 8F–I). Consistently, MI-induced mortality was also lower in *beclin 1*^+/−^ mice than in WT littermate controls (Fig. 8J). In addition, hearts from *beclin 1*^+/−^ mice exhibited reduced fibrotic scars and increased LV wall thickness, and further improved following EMPA treatment (Fig. 8K and 8L). Combined, the unbiast screening demonstrating reduced autophagy through NHE1 knockdown (and EMPA targeting cardiomyocyte NHE1), the *in vitro* studies demonstrating reduced autophagic flux with EMPA treatment, and improvement in myocardial histology and function with reduced autophagy (*beclin 1*^+/−^ mouse), all support a role for EMPA in reducing autosis with glucose deprivation in the ischemic zone.

## DISCUSSION

SGLT2 is mainly expressed in the kidney, and the mechanism of cardiovascular protection of SGLT2 inhibitors remains unclear (Bell and Yellon, 2017; Lytvyn et al., 2017; Packer et al., 2017; Uthman et al., 2018). Treatment of Empagliflozin in diabetic models and WT mice (Xiang et al., 2015) subjected to acute myocardial infarction (Wang et al., 2018) significantly reduced the infarct size, myocardial fibrosis, and significantly improved the survival rate. Proposed hypotheses for the cardiovascular protective mechanisms in diabetics include: (1) inhibition of NHE1, thereby reducing [Na^+^]_c_, [Ca^2+^]_c_ levels and increasing mitochondrial [Ca^2+^]_m_ levels in cardiomyocytes (Baartscheer et al., 2017; Bertero et al., 2018; Uthman et al., 2018); (2) ketone body oxidation (Ferrannini et al., 2016; Lopaschuk and Verma, 2016); (3) ATP production and mitochondrial function (Mudaliar et al., 2016); (4) diuretic effect (Vettor et al., 2017), in addition to many more (Bell and Yellon, 2018). Recently, it has been suggested that induction of autophagy may play a role (Delbridge et al., 2017). However increased autosis is observed with MI (Nah et al., 2020). Prior to these publications we had already set out to use unbiast screening approaches to determine the molecular mechanism for post MI protection by EMPA. We also converged on autophagy but our finding supported reduction rather than induction of autophagy. Autophagy is a clearly a complex and essential process where lack of autophagy as well as excessive autophagy is detrimental. Rescue and protection thus requires regulation of autophagy homeostasis, dependent of the disease process and cellular context.

As a terminally differentiated cell, the death of cardiomyocytes will lead to structural and functional defects in the heart and exacerbate heart failure. Myocardial cell death was classically divided into two major pathways: classical apoptosis and necrosis. However, new cell death patterns such as necroptosis, pyroptosis, ferroptosis and autosis have been discovered (Whelan et al., 2010; Liu et al., 2013; Galluzzi et al., 2018). Among them, autophagy is an evolutionarily conserved intracellular process that mediates organelle turnover, protein degradation, and recirculation of excess and aging or damaged cytoplasmic components in response to a variety of stimuli, including cellular stress, ischemic injury, protein toxicity, infection and nutritional starvation. However, the key role of autophagy in cardiomyocyte survival and its underlying signaling mechanisms are unclear (Lavandero et al., 2015; Bravo-San Pedro et al., 2017; Sciarretta et al., 2018a; Sciarretta et al., 2018b). Whether autophagy is beneficial or harmful in myocardial infarction remains controversial (Liu et al., 2018; Santulli, 2018; Sciarretta et al., 2018c). Inhibition of autophagy (Liu et al., 2018) and activation of autophagy (Xie et al., 2014) have both been reported to reduce myocardial infarct size, and even reverse ventricular remodeling after myocardial infarction and improve cardiac function (Sciarretta et al., 2018c). Autophagy is a needed process but excessive autophagy under conditions of stress may accelerate myocardial cell death (Nah et al., 2020).

With our cultured cardiomyocytes studies, pretreatment with of SGLT2 inhibitors significantly improved the survival of cardiomyocytes response to glucose deprivation (GD) (Fig. 3). GD induces autophagy accompanied by activation of adenylate-activated protein kinase (AMPK) and inactivation of the mammalian target of rapamycin (mTOR). Inhibition of AMPK significantly reduced GD-induced autophagy, but rapamycin-stimulated autophagy did not have an additive effect on GD-induced autophagy, suggesting that AMPK activation and mTOR inhibition independently mediate GD-induced autophagy (Matsui et al., 2007). Our *in vivo* studies demonstrated that myocardial ischemia in mice can induce autophagy, that can be further enhanced during heart reperfusion. This is consistent with a recent study suggesting increased autosis in MI (Nah et al., 2020). Autophagy induced by myocardial ischemia in mice is accompanied by activation of AMPK, whereas autophagy during reperfusion is accompanied by upregulation of the key protein Beclin 1 in the autophagy pathway, but not activation of AMPK. In *beclin 1*^+/−^ mice, induced autophagy and cardiac damage were significantly attenuated during the reperfusion phase. These results indicate that in the heart, ischemia stimulates autophagy via an AMPK-dependent mechanism, whereas ischemia/reperfusion stimulates autophagy via a Beclin1-dependent, AMPK-independent mechanism. Thus, autophagy may play differential roles during ischemia and reperfusion, being protective during ischemia, and detrimental during reperfusion. EMPA serves to reduce the detrimental effects of autosis.

In conclusion, using SGLT2 inhibitors, we provide new mechanistic insights into molecular regulation of autophagy during myocardial ischemia-reperfusion injury. Autophagy is necessary under acute stress to maintain cellular homeostasis by degrading abnormal components within cells, however, excessive autophagy can lead to autosis. In elucidating the target and mechanism for SGLT2 inhibitors, we provide novel targets for management of myocardial ischemia/infarction including support for the development of novel cardiac-specific NHE1 inhibitors as well as autosis inhibitors.

## Electronic supplementary material

Below is the link to the electronic supplementary material.

Electronic supplementary material 1 (PDF 2524 kb)

## References

[CR1] Avogaro A, Fadini GP, Del Prato S (2020). Reinterpreting cardiorenal protection of renal sodium-glucose cotransporter 2 inhibitors via cellular life history programming. Diabetes Care.

[CR2] Baartscheer A, Schumacher CA, Wust RC, Fiolet JW, Stienen GJ, Coronel R, Zuurbier CJ (2017). Empagliflozin decreases myocardial cytoplasmic Na(+) through inhibition of the cardiac Na(+)/H(+) exchanger in rats and rabbits. Diabetologia.

[CR3] Bell RM, Yellon DM (2017). SGLT2 inhibitors: hypotheses on the mechanism of cardiovascular protection. Lancet Diabetes Endocrinol.

[CR4] Bell RM, Yellon DM (2018). SGLT2 inhibitors: hypotheses on the mechanism of cardiovascular protection. Lancet Diabetes Endocrinol.

[CR5] Bertero E, Prates Roma L, Ameri P, Maack C (2018). Cardiac effects of SGLT2 inhibitors: the sodium hypothesis. Cardiovasc Res.

[CR6] Bravo-San Pedro JM, Kroemer G, Galluzzi L (2017). Autophagy and Mitophagy in Cardiovascular Disease. Circ Res.

[CR7] Delbridge LMD, Mellor KM, Taylor DJ, Gottlieb RA (2017). Myocardial stress and autophagy: mechanisms and potential therapies. Nat Rev Cardiol.

[CR8] Ferrannini E, Mark M, Mayoux E (2016). CV protection in the EMPA-REG OUTCOME trial: a “thrifty substrate” hypothesis. Diabetes Care.

[CR9] Galluzzi L, Vitale I, Aaronson SA, Abrams JM, Adam D, Agostinis P, Alnemri ES, Altucci L, Amelio I, Andrews DW (2018). Molecular mechanisms of cell death: recommendations of the Nomenclature Committee on Cell Death 2018. Cell Death Differ.

[CR10] Green JB, Bethel MA, Armstrong PW, Buse JB, Engel SS, Garg J, Josse R, Kaufman KD, Koglin J, Korn S (2015). Effect of Sitagliptin on cardiovascular outcomes in type 2 diabetes. N Engl J Med.

[CR11] Greene SJ, Vaduganathan M, Khan MS, Bakris GL, Weir MR, Seltzer JH, Sattar N, McGuire DK, Januzzi JL, Stockbridge N (2018). Prevalent and incident heart failure in cardiovascular outcome trials of patients with Type 2 diabetes. J Am Coll Cardiol.

[CR12] Hariharan N, Maejima Y, Nakae J, Paik J, Depinho RA, Sadoshima J (2010). Deacetylation of FoxO by Sirt1 plays an essential role in mediating starvation-induced autophagy in cardiac myocytes. Circ Res.

[CR13] Holman RR, Paul SK, Bethel MA, Matthews DR, Neil HA (2008). 10-year follow-up of intensive glucose control in type 2 diabetes. N Engl J Med.

[CR14] Kobayashi S, Xu X, Chen K, Liang Q (2012). Suppression of autophagy is protective in high glucose-induced cardiomyocyte injury. Autophagy.

[CR15] Lavandero S, Chiong M, Rothermel BA, Hill JA (2015). Autophagy in cardiovascular biology. J Clin Invest.

[CR16] Liu CY, Zhang YH, Li RB, Zhou LY, An T, Zhang RC, Zhai M, Huang Y, Yan KW, Dong YH (2018). LncRNA CAIF inhibits autophagy and attenuates myocardial infarction by blocking p53-mediated myocardin transcription. Nat Commun.

[CR17] Liu Y, Shoji-Kawata S, Sumpter RM, Wei Y, Ginet V, Zhang L, Posner B, Tran KA, Green DR, Xavier RJ (2013). Autosis is a Na+, K+-ATPase-regulated form of cell death triggered by autophagy-inducing peptides, starvation, and hypoxia-ischemia. Proc Natl Acad Sci USA.

[CR18] Lopaschuk GD, Verma S (2016). Empagliflozin’s fuel hypothesis: not so soon. Cell Metab.

[CR19] Lytvyn Y, Bjornstad P, Udell JA, Lovshin JA, Cherney DZI (2017). Sodium glucose cotransporter-2 inhibition in heart failure: potential mechanisms, clinical applications, and summary of clinical trials. Circulation.

[CR20] Maack C, Lehrke M, Backs J, Heinzel FR, Hulot JS, Marx N, Paulus WJ, Rossignol P, Taegtmeyer H, Bauersachs J (2018). Heart failure and diabetes: metabolic alterations and therapeutic interventions: a state-of-the-art review from the Translational Research Committee of the Heart Failure Association-European Society of Cardiology. Eur Heart J.

[CR21] Matsui Y, Takagi H, Qu X, Abdellatif M, Sakoda H, Asano T, Levine B, Sadoshima J (2007). Distinct roles of autophagy in the heart during ischemia and reperfusion: roles of AMP-activated protein kinase and Beclin 1 in mediating autophagy. Circ Res.

[CR22] McMurray JJV, Solomon SD, Inzucchi SE, Kober L, Kosiborod MN, Martinez FA, Ponikowski P, Sabatine MS, Anand IS, Belohlavek J (2019). Dapagliflozin in patients with heart failure and reduced ejection fraction. N Engl J Med.

[CR23] Mudaliar S, Alloju S, Henry RR (2016). Can a shift in fuel energetics explain the beneficial cardiorenal outcomes in the EMPA-REG OUTCOME Study? A unifying hypothesis. Diabetes Care.

[CR24] Nah J, Zhai P, Huang CY, Fernandez AF, Mareedu S, Levine B, Sadoshima J (2020). Upregulation of Rubicon promotes autosis during myocardial ischemia/reperfusion injury. J Clin Invest.

[CR25] Nakamura TY, Iwata Y, Arai Y, Komamura K, Wakabayashi S (2008). Activation of Na+/H+ exchanger 1 is sufficient to generate Ca2+ signals that induce cardiac hypertrophy and heart failure. Circ Res.

[CR26] Nassif M, Kosiborod M (2018). Effect of glucose-lowering therapies on heart failure. Nat Rev Cardiol.

[CR27] Nassif ME, Windsor S, Tang F, Khariton Y, Husain M, Inzucchi SE, McGuire D, Pitt B, Scirica BM, Austin B (2019). Dapagliflozin effects on biomarkers, symptoms, and functional status in patients with heart failure with reduced ejection fraction: the DEFINE-HF Trial. Circulation.

[CR28] Neal B, Perkovic V, Mahaffey KW, de Zeeuw D, Fulcher G, Erondu N, Shaw W, Law G, Desai M, Matthews DR (2017). Canagliflozin and cardiovascular and renal events in type 2 diabetes. N Engl J Med.

[CR29] Nissen SE, Wolski K (2007). Effect of rosiglitazone on the risk of myocardial infarction and death from cardiovascular causes. N Engl J Med.

[CR30] Packer M (2017). Activation and inhibition of sodium-hydrogen exchanger is a mechanism that links the pathophysiology and treatment of diabetes mellitus with that of heart failure. Circulation.

[CR31] Packer M (2020). Autophagy stimulation and intracellular sodium reduction as mediators of the cardioprotective effect of sodium-glucose cotransporter 2 inhibitors. Eur J Heart Fail.

[CR32] Packer M (2020). SGLT2 inhibitors produce cardiorenal benefits by promoting adaptive cellular reprogramming to induce a state of fasting mimicry: a paradigm shift in understanding their mechanism of action. Diabetes Care.

[CR33] Packer M, Anker SD, Butler J, Filippatos G, Zannad F (2017). Effects of sodium-glucose cotransporter 2 inhibitors for the treatment of patients with heart failure: proposal of a novel mechanism of action. JAMA Cardiol.

[CR34] Perry RJ, Rabin-Court A, Song JD, Cardone RL, Wang Y, Kibbey RG, Shulman GI (2019). Dehydration and insulinopenia are necessary and sufficient for euglycemic ketoacidosis in SGLT2 inhibitor-treated rats. Nat Commun.

[CR35] Santos-Gallego CG, Requena-Ibanez JA, San Antonio R, Ishikawa K, Watanabe S, Picatoste B, Flores E, Garcia-Ropero A, Sanz J, Hajjar RJ (2019). Empagliflozin ameliorates adverse left ventricular remodeling in nondiabetic heart failure by enhancing myocardial energetics. J Am Coll Cardiol.

[CR36] Santulli G (2018). Cardioprotective effects of autophagy: eat your heart out, heart failure!. Sci Transl Med.

[CR37] Sciarretta S, Forte M, Frati G, Sadoshima J (2018). New insights into the role of mTOR signaling in the cardiovascular system. Circ Res.

[CR38] Sciarretta S, Maejima Y, Zablocki D, Sadoshima J (2018). The role of autophagy in the heart. Annu Rev Physiol.

[CR39] Sciarretta S, Yee D, Nagarajan N, Bianchi F, Saito T, Valenti V, Tong M, Del Re DP, Vecchione C, Schirone L (2018). Trehalose-induced activation of autophagy improves cardiac remodeling after myocardial infarction. J Am Coll Cardiol.

[CR40] Scirica BM, Bhatt DL, Braunwald E, Steg PG, Davidson J, Hirshberg B, Ohman P, Frederich R, Wiviott SD, Hoffman EB (2013). Saxagliptin and cardiovascular outcomes in patients with type 2 diabetes mellitus. N Engl J Med.

[CR41] Taylor SI, Blau JE, Rother KI (2015). Possible adverse effects of SGLT2 inhibitors on bone. Lancet Diabetes Endocrinol.

[CR42] Todd AE, Orengo CA, Thornton JM (2001). Evolution of function in protein superfamilies, from a structural perspective. J Mol Biol.

[CR43] Ueda P, Svanstrom H, Melbye M, Eliasson B, Svensson AM, Franzen S, Gudbjornsdottir S, Hveem K, Jonasson C, Pasternak B (2018). Sodium glucose cotransporter 2 inhibitors and risk of serious adverse events: nationwide register based cohort study. BMJ.

[CR44] Uthman L, Baartscheer A, Bleijlevens B, Schumacher CA, Fiolet JWT, Koeman A, Jancev M, Hollmann MW, Weber NC, Coronel R (2018). Class effects of SGLT2 inhibitors in mouse cardiomyocytes and hearts: inhibition of Na(+)/H(+) exchanger, lowering of cytosolic Na(+) and vasodilation. Diabetologia.

[CR45] Vettor R, Inzucchi SE, Fioretto P (2017). The cardiovascular benefits of empagliflozin: SGLT2-dependent and -independent effects. Diabetologia.

[CR46] Wang D, Hu X, Lee SH, Chen F, Jiang K, Tu Z, Liu Z, Du J, Wang L, Yin C (2018). Diabetes exacerbates myocardial ischemia/reperfusion injury by down-regulation of microRNA and up-regulation of O-GlcNAcylation. JACC Basic Transl Sci.

[CR47] Wang Y, Meyer JW, Ashraf M, Shull GE (2003). Mice with a null mutation in the NHE1 Na+-H+ exchanger are resistant to cardiac ischemia-reperfusion injury. Circ Res.

[CR48] Whelan RS, Kaplinskiy V, Kitsis RN (2010). Cell death in the pathogenesis of heart disease: mechanisms and significance. Annu Rev Physiol.

[CR49] Wiviott SD, Raz I, Bonaca MP, Mosenzon O, Kato ET, Cahn A, Silverman MG, Zelniker TA, Kuder JF, Murphy SA (2019). Dapagliflozin and cardiovascular outcomes in type 2 diabetes. N Engl J Med.

[CR50] Xiang Y, Cheng J, Wang D, Hu X, Xie Y, Stitham J, Atteya G, Du J, Tang WH, Lee SH (2015). Hyperglycemia repression of miR-24 coordinately upregulates endothelial cell expression and secretion of von Willebrand factor. Blood.

[CR51] Xie M, Kong Y, Tan W, May H, Battiprolu PK, Pedrozo Z, Wang ZV, Morales C, Luo X, Cho G (2014). Histone deacetylase inhibition blunts ischemia/reperfusion injury by inducing cardiomyocyte autophagy. Circulation.

[CR52] Yang J, Zhang Y (2015). I-TASSER server: new development for protein structure and function predictions. Nucleic Acids Res.

[CR53] Yurista SR, Sillje HHW, Oberdorf-Maass SU, Schouten EM, Pavez Giani MG, Hillebrands JL, van Goor H, van Veldhuisen DJ, de Boer RA, Westenbrink BD (2019). Sodium-glucose co-transporter 2 inhibition with empagliflozin improves cardiac function in non-diabetic rats with left ventricular dysfunction after myocardial infarction. Eur J Heart Fail.

[CR54] Zelniker TA, Braunwald E (2018). Cardiac and renal effects of sodium-glucose co-transporter 2 inhibitors in diabetes: JACC State-of-the-Art Review. J Am Coll Cardiol.

[CR55] Zelniker TA, Wiviott SD, Raz I, Im K, Goodrich EL, Bonaca MP, Mosenzon O, Kato ET, Cahn A, Furtado RHM (2019). SGLT2 inhibitors for primary and secondary prevention of cardiovascular and renal outcomes in type 2 diabetes: a systematic review and meta-analysis of cardiovascular outcome trials. Lancet.

[CR56] Zhang J, Ney PA (2009). Role of BNIP3 and NIX in cell death, autophagy, and mitophagy. Cell Death Differ.

[CR57] Zheng SL, Roddick AJ, Aghar-Jaffar R, Shun-Shin MJ, Francis D, Oliver N, Meeran K (2018). association between use of sodium-glucose cotransporter 2 inhibitors, glucagon-like peptide 1 agonists, and dipeptidyl peptidase 4 inhibitors with all-cause mortality in patients with type 2 diabetes: a systematic review and meta-analysis. JAMA.

[CR58] Zinman B, Wanner C, Lachin JM, Fitchett D, Bluhmki E, Hantel S, Mattheus M, Devins T, Johansen OE, Woerle HJ (2015). Empagliflozin, cardiovascular outcomes, and mortality in type 2 diabetes. N Engl J Med.

