# Supplementary material for: Cardioprotective mechanism of SGLT2 inhibitor against myocardial infarction is through reduction of autosis
Source: Protein Cell. 2021 Jan 8;13(5):336–59. doi: 10.1007/s13238-020-00809-4 (PMC9008115; doi:10.1007/s13238-020-00809-4)
Supplement: Supplementary file 1 — Electronic supplementary material 1 (PDF 2524 kb) [file 13238_2020_809_MOESM1_ESM.pdf]

Materials and Methods

Animal study.

All mouse studies were approved by Tongji University Animal Research Committee. Wildtype (WT) (C57BL/6J background) and diabetic mice (BKS.Cg-Dock7m1/1 Leprd/b/j) were purchased from the Shanghai SLAC Laboratory and the Gempharmatech. Streptozotocin (STZ)-induced diabetic mice in the C57BL/6J background were injected with STZ (50 mg/kg) intraperitoneally for 5 consecutive days or 150mg/kg for one time to induce recurrent episodes of acute hyperglycemia. Heterozygous Beclin-1 knock-out (*beclin 1*$^{+/-}$) mice (C57BL/6J background) were bred in-house and purchased from the Gempharmatech.

Generation of Nhe1 KO mice.

NHE1 KO mice （*Nhe1*$^{-/-}$） were generated using CRISPR/Cas9 system and were performed by Tongji University and BIOCYTOGEN. The brief process is as follows: sgRNA was transcribed in vitro. Cas9 mRNA and sgRNAs were microinjected into fertilized embryos of C57BL/6N mice. Fertilized eggs were transplanted to obtain positive F0 mice which were confirmed by PCR and sequencing. A stable F1 generation mouse model was obtained by mating positive F0 generation mice with C57BL/6N mice. Chimeric mice were generated and bred with C57BL6/N females and germline transmission was identified by PCR analysis. PCR genotyping analysis of Nhe1 KO mice was carried out using genomic DNA from tail biopsies and the following primers (Figure 6J): 1. WT - Forward primer: 5'-TTCTCCTGTCTCTCTCACATCCCCG-3'; WT - Reverse primer:

5'-GGAGCCAGAAGAACAGAAGGTTGGG-3'; 2. WT - Forward primer: 5'-TTCTCCTGTCTCTCTCACATCCCCG-3'; Mut - Reverse primer: Mut-5'-ATGCCTCCACTTCAAGAGTGCCATC-3'. The reaction mix was composed of 10 μL 2xTaq Plus Master Mix (Dye Plus) (New England Biolabs), 8.6 μL H_2O, 1 μL DNA, using the following amplification protocol (95 °C 5 min; 95 °C 30 s; 61 °C 30 s; 72 ℃ 1 min; 30 cycles; 72 ℃ 10 min). Amplification product sizes were as follows: wild type (643 bp), *Nhe1*$^{+/-}$ (643 bp and 444 bp), and *Nhe1*$^{-/-}$ (444 bp)

In vivo myocardial I/R and TTC staining

WT were anesthetized by spontaneous inhalation and maintained under general anesthesia with 1–2% isoflurane and subjected to left coronary artery occlusion for 30 min, followed by 3 h /24 h of reperfusion. Hearts were then excised and stained with Evans blue and triphenyl tetrazolium chloride to measure the ischemic area at risk and the area of necrosis, respectively.

In vivo myocardial infarction and Masson staining

Mice were anesthetized by spontaneous inhalation and maintained under general anesthesia with 1–2% isoflurane. Animals were mechanically ventilated using a rodent ventilator (Harvard Apparatus) connected to an endotracheal tube. The heart was exposed by a left side limited thoracotomy and the LAD was ligated with a 6-0 polyester suture 1 mm from the apex of the normally positioned left auricle and samples were analyzed 7 days after injury. Tissue from the left ventricle fixed in 4% paraformaldehyde were embedded in opti-mum cutting temperature compound (OCT) and cut into 10μm thick sections. The sections were stained separately with Masson’s

trichrome. The morphology of the cardiomyocytes and the deposition of collagen were observed by microscope.

Echocardiography

Echocardiography was conducted on patients using an iE33 Doppler ultrasonography system (Philips) in this study. The images were obtained with the subjects at rest and lying in the lateral decubitus position. Standard echocardiographic studies, including left ventricular maximal wall thickness, left atrial and left ventricular dimensions at end-systole and end-diastole, septal thickness and posterior wall thickness in end-diastole, were performed according to previously published methods. The heart was imaged in the two-dimensional parasternal short-axis view, and an M-mode echocardiogram of the midventricle was recorded at the level of the papillary muscles. Heart rate, intraventricular septum and posterior wall thickness, and end-diastolic and end-systolic internal dimensions of the left ventricle were obtained from the M-mode image.

Cell culture and Isolation of neonatal rat cardiomyocytes

H9c2 rat cardiomyoblast cells were obtained from ATCC and cultured in high glucose (4,500 mg/l) Dulbecco's modified Eagle's medium (DMEM) supplemented with 10%FBS. Cardiomyocytes were isolated from neonatal Wistar rats (1 to 2 days). In brief, after dissection, hearts were washed and minced in phosphate buffer saline. Tissues were then dispersed in a series of incubations at 37 ℃ in 1.2 mg/ml pancreatin and 0.14 mg/ml collagenase. Subsequent supernatants were collected and centrifuged at 200 g for 5 min. After centrifugation, cells were resuspended in

Dulbecco's modified Eagle medium/F-12 (11330032, Gibco) containing 5% heating activated horse serum, 0.1 mmol/l ascorbate, insulin-transferring-sodium selenite media supplement, 100U/ml penicillin, 100 mg/ml streptomycin, and 0.1 mmol/l bromodeoxyuridine. The dissociated cells were pre-plated at 37 ℃ for 1 h. The cells were then diluted to 1x 10^6 cells/ml and plated in 10 mg/ml laminin-coated different culture dishes according to the specific experimental requirements.

Membrane Transporter/Ion Channel Compound library screen

A small-molecule compound library with 387 candidates (HY-1011 , HY-LD-000001445 , MCE), targeting membrane transporters and ion channels, was used for pharmacological screening with cardiomyocytes protection of anti-glucose deprivation (GD). Cultured cardiomyocytes were incubated with each of candidate compound at concentration of 10μM or 100μM prior to GD for 24 hours, viability of cardiomyocytes were determined at 450nm using the Cell Counting Kit-8 (MCE). Duplicate treatment were averaged and normalized to control from the same plate. Normalized values were then processed by calculating the relative fold change (compound/control) for each compound at the dosage. We then obtained the compounds that were more than 1.5 fold different for further analysis according to their target specificity.

Measuring pH changes with BCECF-AM and Na^+ changes with SBFI AM

Intracellular pH measurements were performed as described previously. Cell staining was performed in Hanks' Balanced Salt Solution (14025-092, gibco) containing 5 μM BCECF AM (S1006, Beyotime) in the presence of 0.07% Ammonia solution

(80006761, Sinopharm Chemical Reagent) for 30min at 37℃, 5% CO2 in the darkness. After staining, cells were washed twice for 5 min in HBSS. BCECF-AM fluorescence (excitation, 488 nm; emission, >515 nm) was detected using TECAN Infinite M200 Pro Multi-Mode Microplate Reader (30080303, TECAN) and fluorescence microscope (Leica). Cells were stained with 300 nm DAPI, a fluorescent marker of cell nuclei (Thermo), to determine total cell counts. Intracellular Na^+ measurements were performed as described previously. Cell staining was performed in Hanks' Balanced Salt Solution (14025-092, gibco) containing 5 μM SBFI AM (S1148, sigma) in the presence of 0.1% Pluronic F-127 for 2-4h at 37℃, 5% CO2 in the darkness. After staining, cells were washed twice for 5 min in HBSS. SBFI AM fluorescence (excitation, 340 nm; emission, >500 nm) was detected using Tecan Infinite M200 Pro Multi-Mode Microplate Reader (30080303, TECAN).

Contraction Force Measurements

Neonatal rat cardiomyocytes were seeded on confocal dishes 2-3 days before and measured in their normal culture medium with or without EMPA at 2, 4, 12, 24h in an environment at 37℃. Video-based motion edge detection system was used to assess cellular contractility of contracting CMs. Briefly, CMs were visualized using a Zeiss CFM-500 inversion fluorescence microscope coupled to FelixGX video microscopy system software (PTI). Spontaneous contraction traces were recorded and the resting cell length and the peak cell shortening amplitude of each cell were determined using commercially available data analysis software (FelixGX, PTI). Only isolated cardiomyocytes with appropriate morphology and function were used, and

non-shortening cells were excluded from analysis.

Western blotting

The cardiac cells (H9c2 and neonatal rat cardiomyocytes) and mouse heart tissue were homogenized, and lysed in RIPA buffer containing protease and phosphatase inhibitors (Sigma). Proteins were electrophoresed and separated by SDS-PAGE and transferred to polyvinylidene difluoride membranes (Millipore). The membranes were blocked in 5% BSA and incubated with antibodies of LC3II/I (4108S, CST), P62 (5114, CST), NHE-1 (ab67314, Abcam), GAPDH (2118S, CST), β-tubulin (2146, CST) and Beclin1 (ab207612, Abcam) overnight at 4 ℃. The membranes were incubated with appropriate secondary antibodies conjugated to horseradish peroxidase and subjected to immunoblot analysis with Image Lab Software (Bio-Rad).

Immunofluorescence staining

Cells and cardiac tissue sections were fixed with 4% paraformaldehyde, permeabilized with 0.05% Triton X-100, blocked with 1% BSA, and incubated with appropriate primary antibodies for 24h at 4 ℃. The samples were then incubated with Alexa Fluor 594 or 488 conjugated secondary antibodies (Invitrogen) at 37 ℃ for 1-2h and subsequently counterstained with DAPI. Neonatal rat cardiomyocytes were validated by immunostaining of α-actinin (Abcam). For characterization of mouse tissue sections, immunofluorescent staining were performed using antibodies for sarcomeric α-actinin (Abcam), ki67 (Abcam) and LC3 (Abcam)). Labeled cells or cardiac tissue sections were examined and imaged with a fluorescence microscope (Leica). Cell surface area size was quantified using the ImageJ software package.

Quantitative RT–PCR

Total RNA was isolated from the left ventricle or cultured cardiomyocytes for analysis using the miRNeasy Mini Kit (1038703, QIAGEN). The mRNA levels were determined by quantitative RT–PCR. For reverse transcription and amplification, we used iScript Reverse Transcription Supermix for RT-qPCR (1708841, BIO-RAD) and the SsoFast EvaGreen Supermix (1725201, BIO-RAD), respectively. The PCR primers were obtained from Sangon (Table S3). We constructed quantitative PCR standard curves using the corresponding complementary DNA, and all data were normalized to Gapdh mRNA content.

HPLC analysis

HPLC analysis was performed with different types of sample. HPLC was performed on an Agilent 1260 (Agilent) equipped with a Waters C18 (5 μm, 4.6 * 250 mm) (Waters). Elution of EMPA was performed with 35% acetonitrile in water for 20 min at a flow rate of 1 ml/min. Samples were analyzed at specific absorption wavelength of 230 nm. Elution of tamoxifen was performed with 1% triethylamine (PH 8.5) in water for 20 min at a flow rate of 1 ml/min. Samples were analyzed at specific absorption wavelength of 250 nm.

Sample Preparation was obtained from H9c2-NHE1-KO cell or the neonatal rat cardiomyocytes that had been treated with 100μM Empagliflozin or 10μM Tamoxifen for 24h or 48h. After a period of time, the cell culture medium is treated as extracellular fluid. Then cells were washed with phosphate-buffered saline and trypsinized, neutralized with complete medium. The suspension was repeatedly frozen

and thawed three times. The intracellular fluid was obtained by centrifugation at 16000g for 5 minutes. The sediment is added to the cell membrane lysate, incubated on ice for 5 min, and centrifuged at 8000g for 5 min to form the cell membrane fraction. The three components of cells were subjected to HPLC analysis.

RNA-seq analysis.

The quality of the reads was evaluated with Fast QC. The samples information of clean data was shown in Tab S1. The paired-end clean RNA-seq reads were aligned to the mouse reference Ensembl Version GRCm38.92 using the splice-aware aligner STAR (v2.4.0j). Browser views of gene tracks and peaks were shown using Integrated Genomics Viewer (IGV), wherein genes were from the Illumina Genomes gene annotation with UCSC data source for mm10. The abundance of each gene was quantified as TPM (Transcripts per million) value, which was evaluated by a statistical method RSEM (RNA-Seq by Expectation Maximization). RSEM uses a generative model of RNA-seq reads and the EM algorithm, taking read mapping uncertainty into account and achieving the most accurate abundance estimates. We calculated the standard deviation (SD) of each gene over 4 samples, a wildtype (*Nhe1*$^{+/+}$) mouse (WT), two heterozygous (*Nhe1*$^{+/-}$) mice as well as mutant (*Nhe1*$^{-/-}$) mouse, and used the gene expression profiles of such samples with SD >= 0.5 genes to generate a hierarchical clustering and heatmap with the “pheatmap” package in R. The analysis of18 differentially expressed genes (DEGs) were performed using the GFOLD, which generalizes the fold change by considering the posterior distribution of log fold change and is useful when no replicate is available. DEGs were further

mapped onto the Gene Ontology (GO) as well as Kyoto Encyclopedia of Genes and Genomes (KEGG), and the adjusted p value indicating whether a function was enriched by DEGs was calculated using the hypergeometric distribution.

Molecular Docking

A homology model of the protein structure of NHE-1 was prepared using a human NHE1 protein structure （from UniProt (https://www.uniprot.org/) with ID: P19634） as a template. A molecular docking study was carried out using the following method to explore the possible interaction between NHE and SGLT2i. (a) Since homology below 30% sequence identity limits single-sequence based searches, the molecular modelling was carried out by Iterative Threading ASSEmbly Refinement (I-TASSER) server (http://zhanglab.ccmb.med.umich.edu/I-TASSER), an integrated platform for automated protein structure and function prediction based on the sequence-to-structure-to-function paradigm (J. Yang, et al. Nucleic acids research, 2015). (b) The structural refinement of the generated NHE1 model from I-TASSER was performed through EM and MD simulation in explicit aqueous environment using GROMACS (M. J. Abraham, et al. SoftwareX, 2015). (c) MOTIF Finder (http://www.genome.jp/tools/motif/) was used to ascertain the probable motifs present in the protein by analysis the amino acid sequences. E-value reveals the significance of the hit. (d) DoGSiteScorer (https://proteins.plus/), an automated pocket detection and analysis tool, was used for the identification of surface pockets and sub-pockets for catalytic domain of modeled NHE1 structure protein as well as druggability assessments using support vector machine (SVM) (A. Volkamer, et al. Journal of

chemical information and modeling, 2012). (e) The prediction of possible ligand binding residues of NHE1 was generated using COACH (http://zhanglab.ccmb.med.umich.edu/COACH/). (f) Afterwards, the interactions of Empaglifozin, Dapagliflozin, Canagliflozin and Cariporide with NHE1 were studied using molecular docking by Genetic optimization for Ligand Docking (GOLD, version 5.3.0) to predict the conformation and binding affinity of these small molecules towards NHE1 (M. L. Verdonk, et al. Journal of medicinal chemistry, 2015). Based on the interaction studies, Empaglifozin, Dapagliflozin and Canagliflozin were selected as the hit molecules for further analysis in the wetlab.

Statistical analysis. Results are shown as the mean ± S.D. Paired data were evaluated using a Student’s t-test. A one-way analysis of variance with the Bonferroni post hoc test was used for multiple comparisons. The Kaplan–Meier method with a log-rank test was used for survival analysis. Data were analyzed using GraphPad Prism 7.

Figure S1

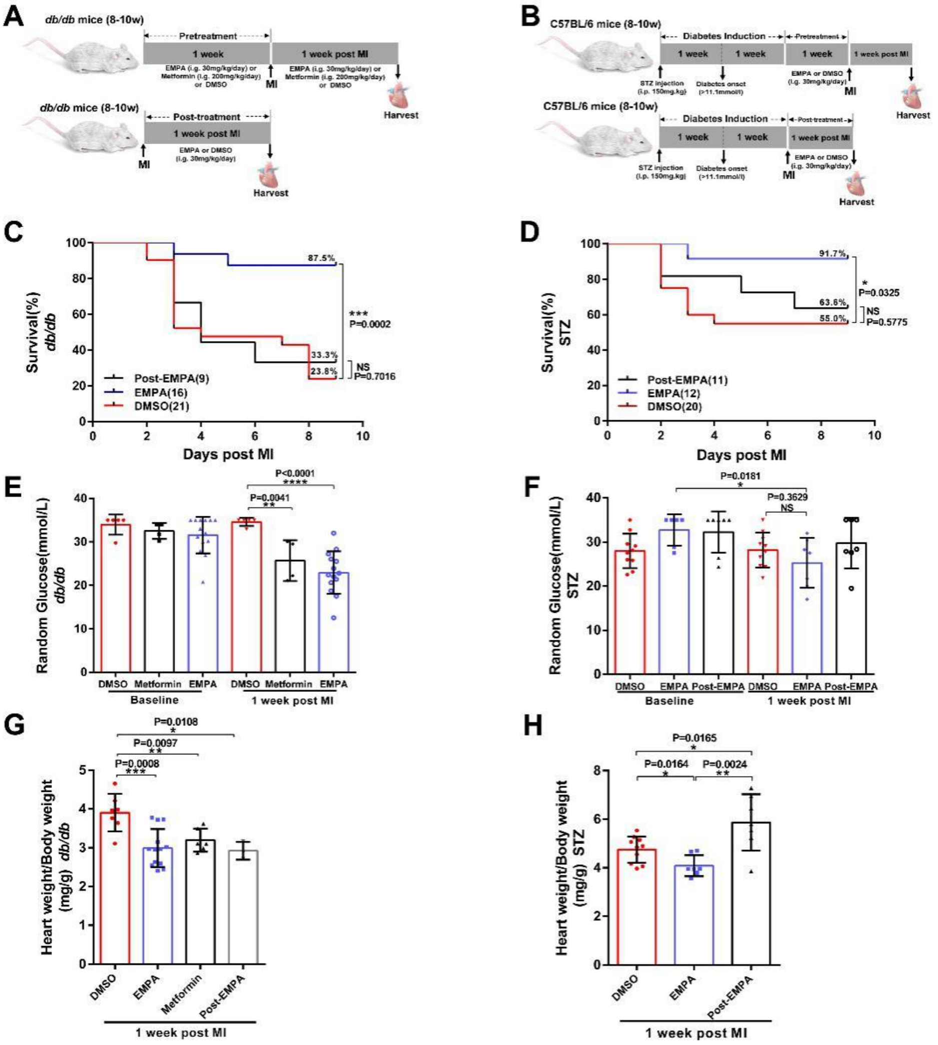

(A, B) Schematic of experimental design of EMPA delivery and MI surgery on *db/db* and STZ-induced diabetic mice (T1DM). (C, D) Survival curve of *db/db* and T1DM (with pretreatment, post-treatment EMPA or DMSO) mice subjected to ligation of left anterior descending coronary artery (LAD) followed by observation for 1 week. (E) Random glucose of *db/db* mice response to EMPA and metformin. (F) Random glucose of T1DM mice response to EMPA and post-treatment EMPA. (G, H) A comparison of EMPA and other groups on heart/body weight on *db/db* and T1DM mice post MI. All date are presented as mean ±S.D, *P<0.05, **P<0.01, ***P<0.005.

Table S1

Echocardiographic Parameters for *db/db* mice.

db/db mice		HR (bpm)	LVIDd (mm)	LVPWd (mm)	LVIDs (mm)	LVPWs (mm)	EF (%)	FS (%)
Baseline	DMSO	415.0±32.7	3.5±0.4	1.0±0.2	2.3±0.4	1.4±0.2	65.1±9.0	65.1±9.0
	EMPA	409.8±22.1	3.6±0.3	0.9±0.1	2.4±0.5	1.3±0.2	62.9±11.0	62.9±11.0
1 week post MI	DMSO	$351.7±17.7^{**}$	$4.6±0.7^{**}$	1.2±0.5	$3.9±0.7^{***}$	1.3±0.4	$31.1±4.6^{***}$	$14.6±2.3^{***}$
	EMPA	$394.6±14.1^{\#}$	4.2±0.8	1.1±0.3	$3.4±0.7^{*}$	1.2±0.4	$42.1±3.6^{**\#\#}$	$20.5±1.8^{**\#\#}$

Notes: Data are mean ± S.D. *P<0.05, **P<0.01, ***P<0.001 versus baseline-groups. #P<0.05, ##P<0.01 versus DMSO-operated controls.

HR, heart rate; LVIDd, left ventricular internal diameter at end-diastole; LVPWd, posterior wall during diastole; LVIDs, left ventricular internal diameter at end-systole; LVPWs, posterior wall during systole; EF, ejection fraction; FS, fractional shortening.

Figure S2

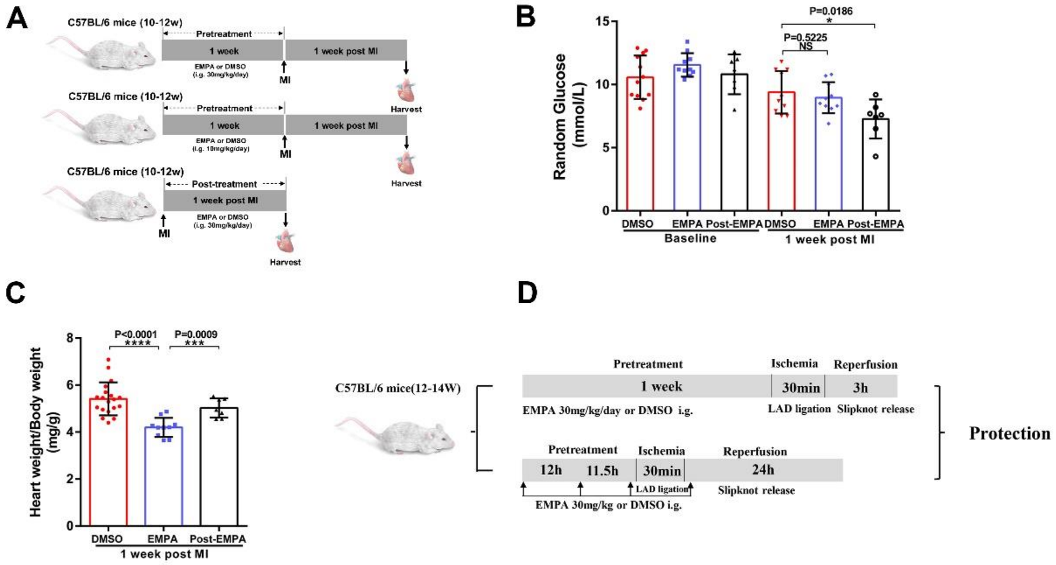

(A) Schematic protocol of EMPA delivery and MI surgery on WT mice. (B) Random glucose of mice response to EMPA and post-treatment EMPA. (C) A comparison of EMPA and other groups on heart/body weight on WT mice post MI. (D) Schematic of experimental design to determine the best suited EMPA delivery way in order to rescue the damage from I/R surgery. All date are presented as mean ±S.D, *P<0.05, **P<0.01, ***P<0.005.

Table S2

Echocardiographic Parameters for C57BL/6 mice.

C57BL/6		HR (bpm)	LVIDd (mm)	LVPWd (mm)	LVIDs (mm)	LVPWs (mm)	EF (%)	FS (%)
Baseline	DMSO	411.1 ± 68.7	3.3 ± 0.3	0.9 ± 0.2	2.0 ± 0.3	1.3 ± 0.2	71.3 ± 6.4	39.8 ± 5.2
	EMPA	408.9 ± 61.6	3.4 ± 0.3	0.7 ± 0.1	2.1 ± 0.3	1.2 ± 0.1	68.4 ± 6.3	37.6 ± 5.0
1 week post MI	DMSO	426.1 ± 53.2	$4.7 \pm 0.6^{***}$	0.7 ± 0.1	$4.1 \pm 0.5^{***}$	$0.8 \pm 0.2^{***}$	$28.0 \pm 5.4^{***}$	$13.1 \pm 2.7^{***}$
	EMPA	387.3 ± 57.4	$4.1 \pm 0.5^{***\#}$	0.7 ± 0.3	$3.3 \pm 0.7^{***\#}$	$0.9 \pm 0.3^{*}$	$42.2 \pm 14.5^{***\#}$	$21.0 \pm 8.2^{***\#}$

Notes: Data are mean ± S.D. *P<0.05, **P<0.01, ***P<0.001 versus baseline-groups. #P<0.05, ##P<0.01 versus DMSO-operated controls.

HR, heart rate; LVIDd, left ventricular internal diameter at end-diastole; LVPWd, posterior wall during diastole; LVIDs, left ventricular internal diameter at end-systole; LVPWs, posterior wall during systole; EF, ejection fraction; FS, fractional shortening.

Figure S3

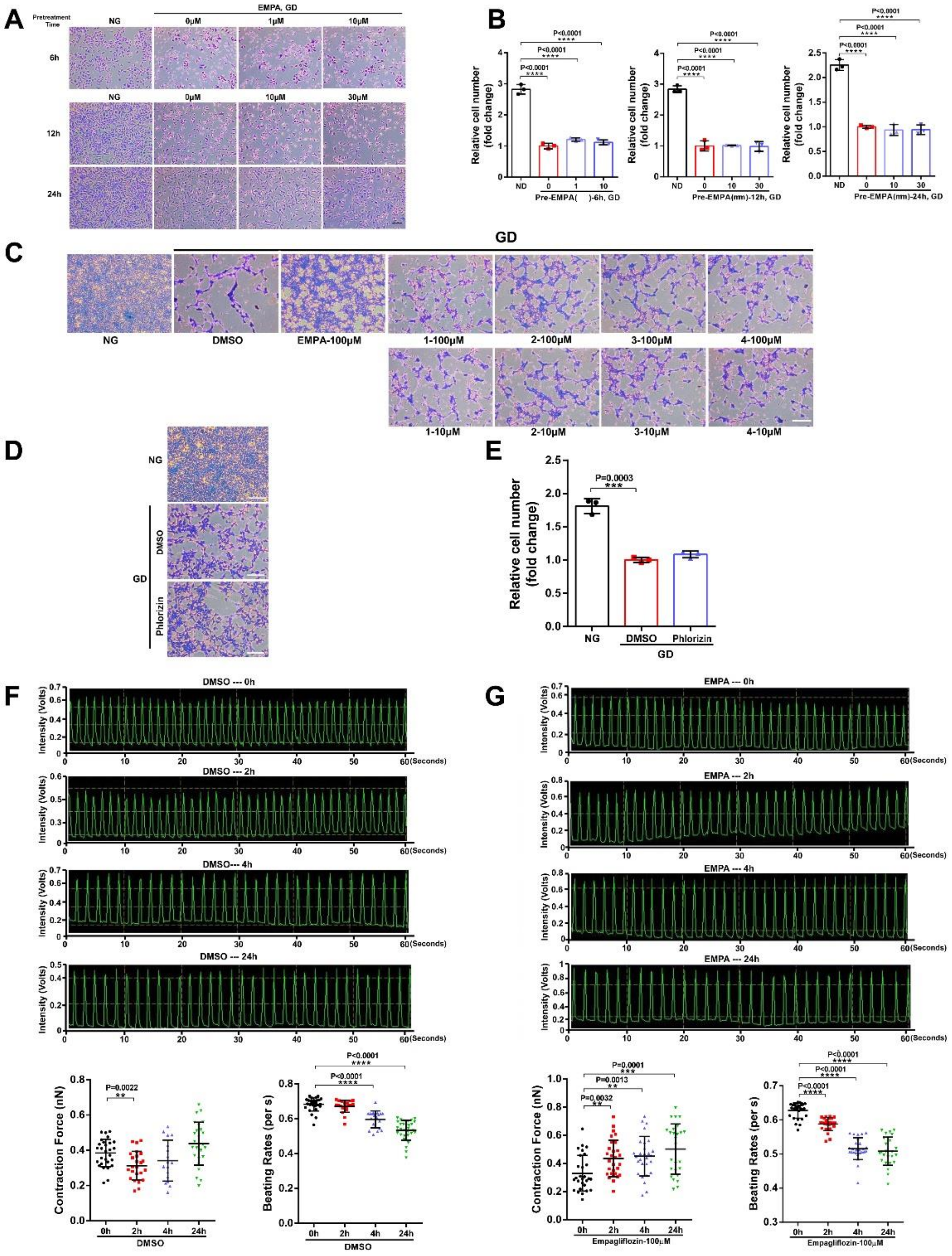

(A) Representative cultured H9c2 pretreated by EMPA during 6, 12, 24h exposed to GD for 24 hours. (B) Quantitation of cell number. (C) Representative images of effects of EMPA major metabolites on H9c2 exposed to GD. (D) Effects of SGLT inhibitor, Phlorizin, on H9c2 exposed to GD and quantitation of cell number (E). (F, G) Representative images showing the contraction force curve measured by FelixGX detection system in cardiomyocytes treated by DMSO and EMPA at 0, 2, 4, 24 hours. And quantification of contraction forces in cardiomyocytes (n>20 in cardiomyocytes from two lines in each group). Data are shown as mean ±S.D. *P<0.05, **P<0.01, ***P<0.005.

Figure S4

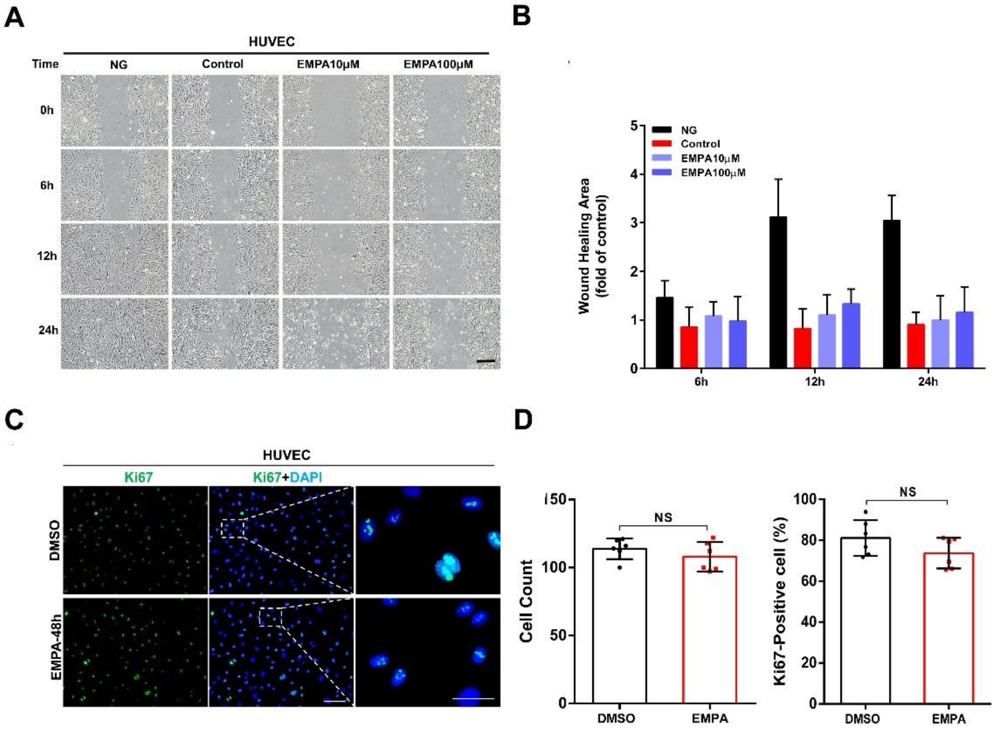

(A) Wound healing assay (scale bar, 50μm) revealed that EMPA treatment could suppress the compromised migratory ability of HUVECs. NG: Normal complete medium. Control: Serum-free medium. n=4 per group. (B) Quantification of the wound healing area. (C) Immunofluorescence staining against Ki67 was performed to assess the proliferative capacity of HUVECs. n=6 per group. Scale bar, 50μm and 500μm. (D) Quantification of the cell count and the cell number of ki67-positive cells. Data are shown as mean ±S.D. *P<0.05, **P<0.01, ***P<0.005.

Figure S5

A

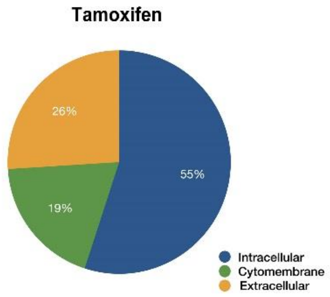

B

Location/μM	Tamoxifen	Empagliflozin
Extracellular	1.4025	71.65
Intracellular	2.9533	3.82
Cytomembrane	1.0365	10.83

C

D

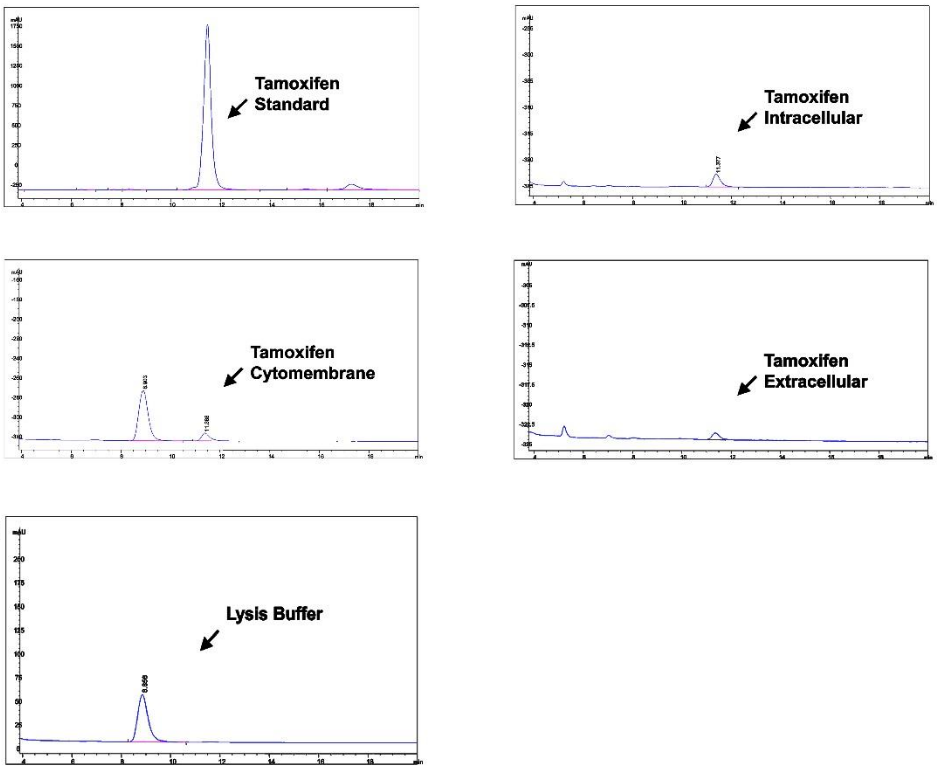

(A) Diagrammatic representation of the tamoxifen proportion of extracellular fluid, intracellular fluid and cell membrane on neonatal rat cardiomyocytes that were treated with tamoxifen. (B) Compare the distribution of tamoxifen and EMPA in the cell components. (C) HPLC analysis of the tamoxifen from the neonatal rat cardiomyocytes of extracellular fluid, intracellular fluid and cytomembrane. (D) HPLC analysis of the cytomembrane lysis buffer.

Figure S6

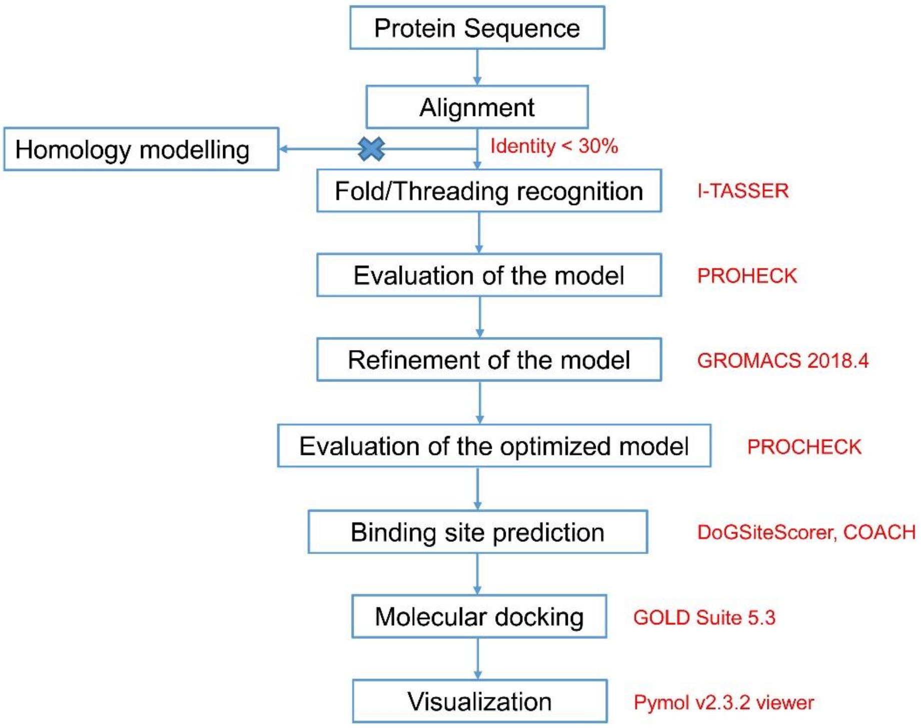

The flowchart for alignment, homology modeling, fold recognition, evaluation and optimization of model, molecular docking and visualization

Figure S7

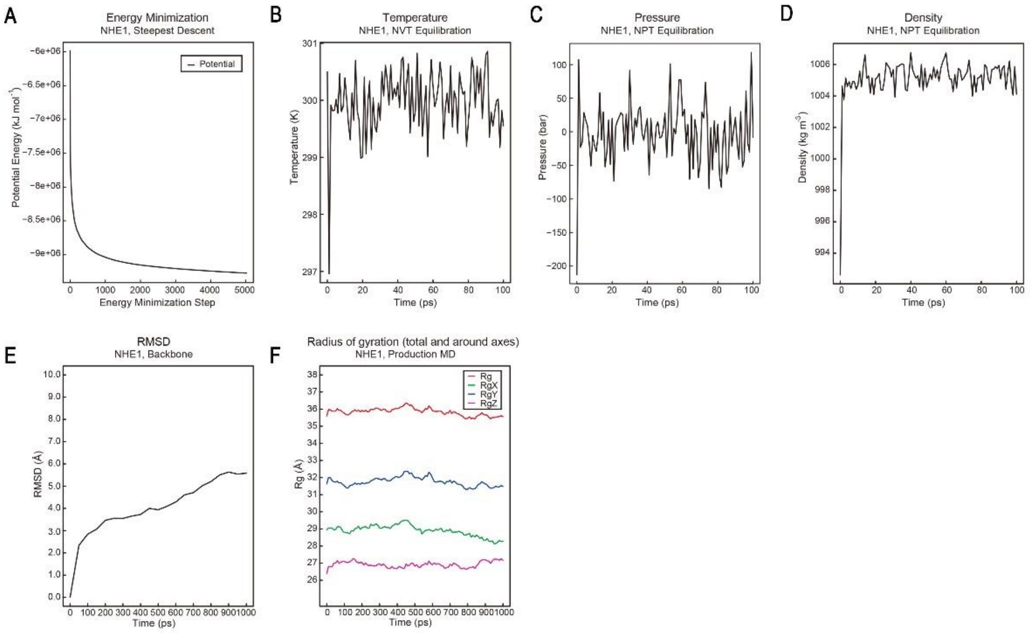

Refinement of the NHE1 model generated from I-TASSER. (A) Potential energy of the NHE1 molecular system. (B) Temperature over the NVT equilibration. (C) Pressure over the NPT equilibration. (D) Density over the NPT equilibration. (E) Backbone RMSD of 1,000 ps simulation. (F) Radius of gyration of 1,000 ps simulation.

Figure S8

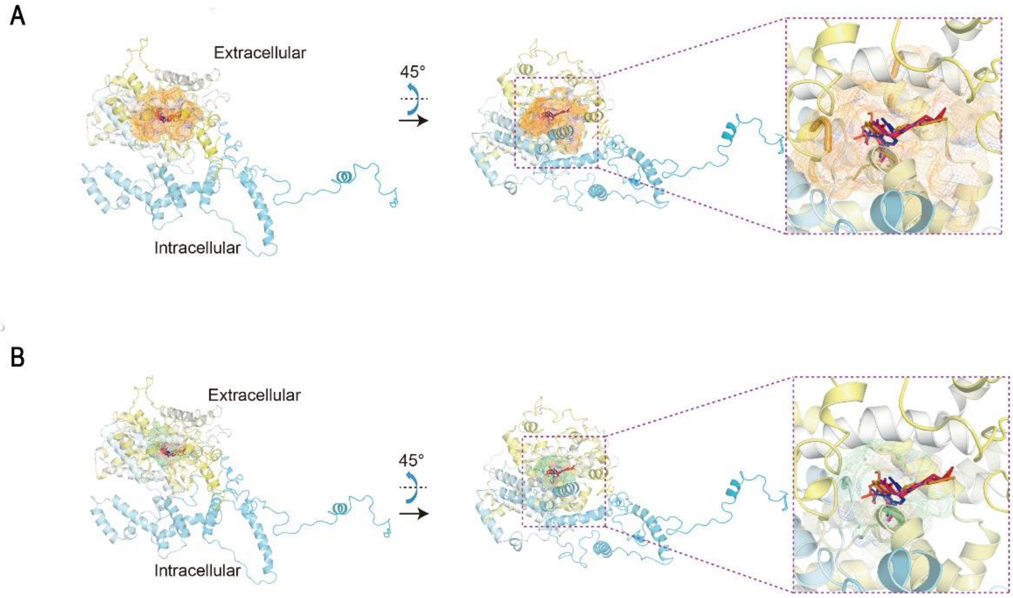

Binding pocket P1 (A) and P1SP00 (B) with SGLT2 inhibitor in NHE1 model. Cariporide is shown in blue, CANA is shown in orange, DAPA in purple, and EMPA in red.

Figure S9

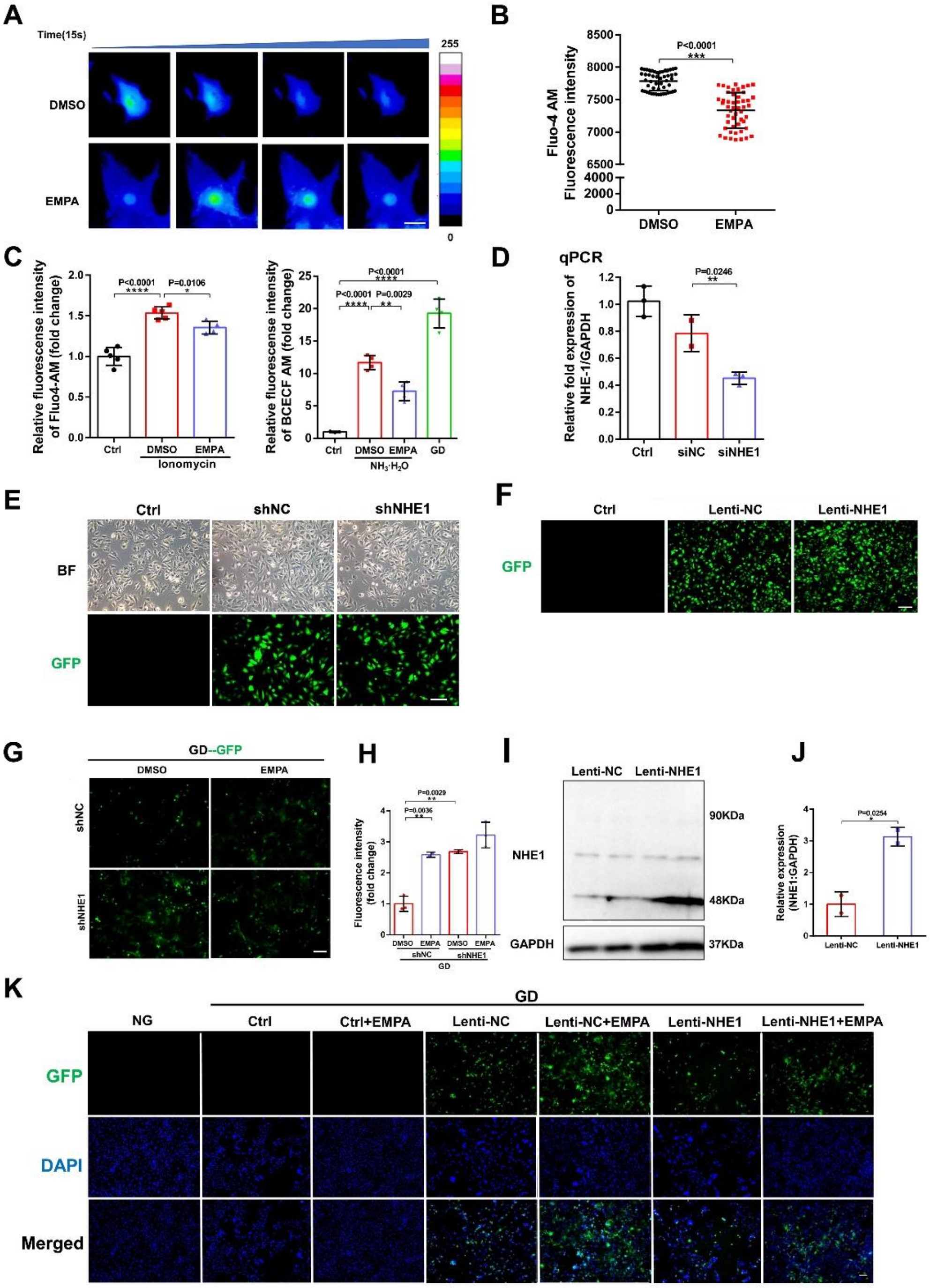

(A) Neonatal rat cardiomyocytes were treated by DMSO and EMPA (100μM) in 30 min. Fluorescent Fluo-4 AM images were taken by a confocal laser microscope. (B) Quantification of average intracellular Ca^{2+} concentration in neonatal rat cardiomyocytes. (C) Effects of EMPA treatment on intracellular Ca^{2+} in ionomycin (5μM) condition treatment and intracellular pH in $NH_3 \cdot H_2O$ condition treatment in neonatal rat cardiomyocytes. (D) Knocking down efficiency of siNHE1 in 293ft evaluated by qPCR. (E, F) Representative images of lentiviral packaging interfering RNA with NHE1 and overexpressing NHE1 the efficiency of infected cardiomyocytes. (G) Representative images for GFP (shNC and shNHE1) on Lenti-transduced H9c2 treated with

EMPA exposed to GD for 24 hours. (H) Quantification of Fluorescence intensity data showing cardiac protection efficiency from shNHE1-transduced H9c2. (I) Western blot for NHE1 and GAPDH expression in Lenti-NC or Lenti-NHE1 H9c2 cells. Quantification of expression level of NHE1 after overexpression was shown in (J). (K) Representative images for GFP (Lenti-NC and Lenti-NHE1) on Lenti-transduced H9c2 treated with EMPA exposed to GD for 24 hours. Data are shown as mean ±S.D. *$P<0.05$, **$P<0.01$, ***$P<0.005$.

Figure S10

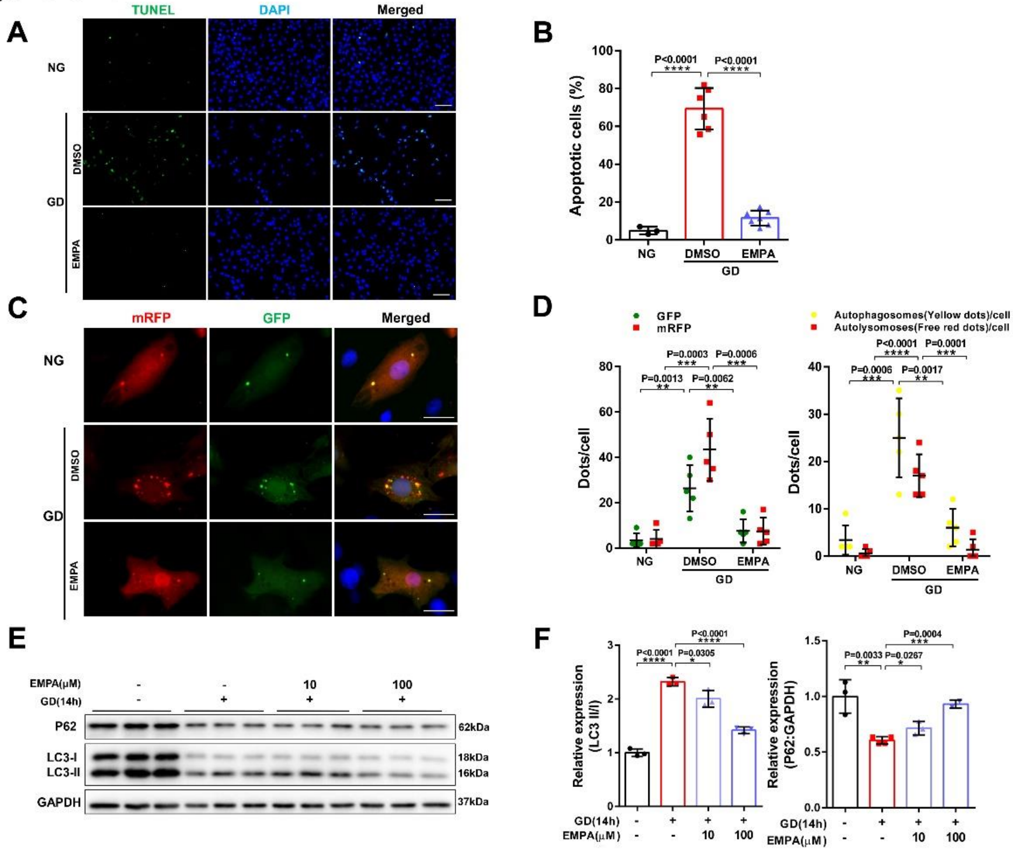

(A) GD-induced H9c2 cell death was examined by TUNEL assay and DAPI staining. Green spots represent apoptotic bodies and blue spots represent cell nuclei. (B) Quantification of the percentage of apoptotic cells based on the total number of cells stained by DAPI, which was rescued by EMPA. (C) H9c2 were infected with an adenovirus expressing mRFP-GFP tandem fluorescent-tagged LC3, cultured with or without EMPA and exposed to GD for 24 hours. (D) The numbers of fluorescent puncta (GFP or mRFP dots) were counted manually from at least three independent experiments. (E, F) Western blot for p62, LC3II/I and GAPDH expression in H9c2. Data in the bar graphs are means ±S.D. *P<0.05, **P<0.01, ***P<0.005.

Figure S11

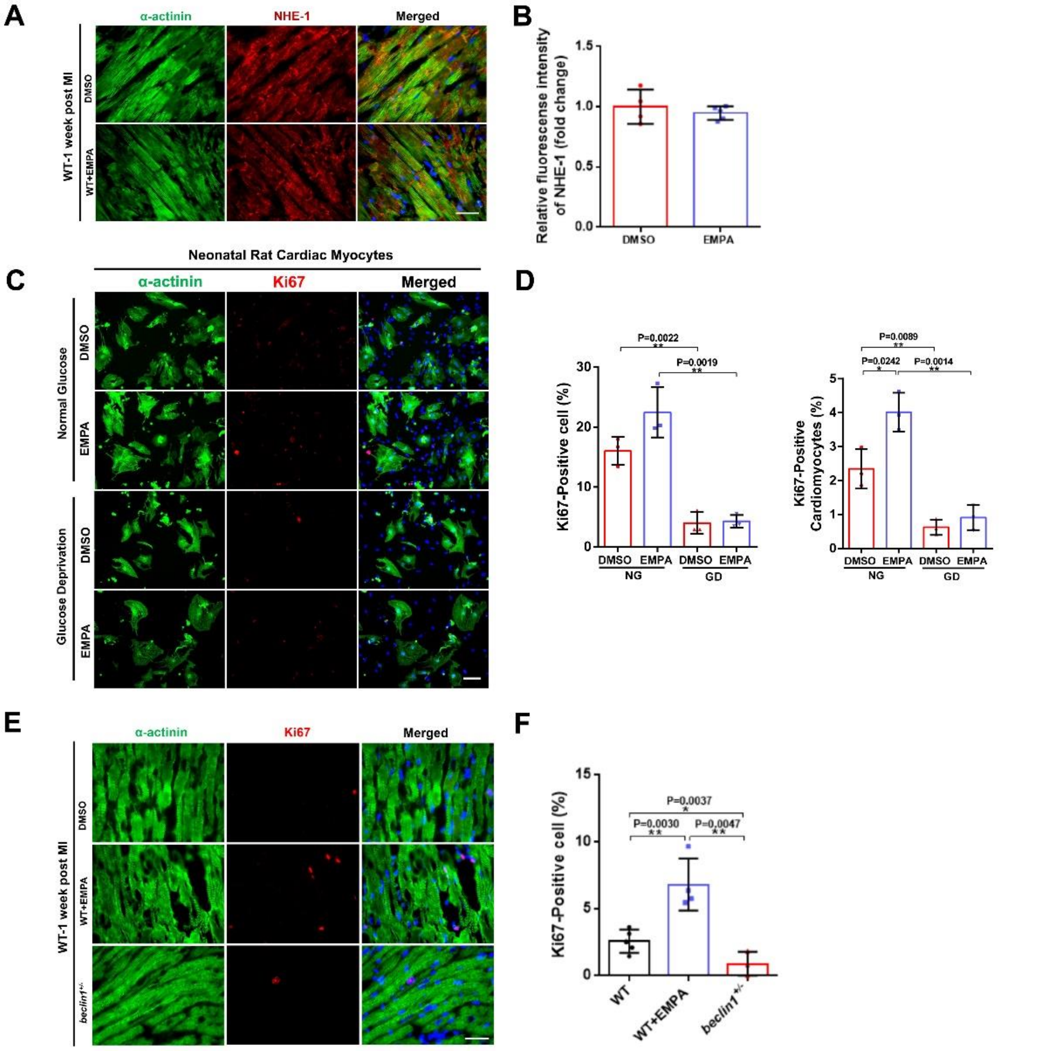

(A, B) Staining of NHE1 in WT mice heart post MI response to EMPA. Quantification was shown in (B). (C) IF staining against Ki67 was performed to assess the proliferative capacity of neonatal rat cardiomyocytes with complete medium or GD for starvation. n=3 per group. Scale bar, 100μm. (D) Quantification of the percentage of Ki67-positive cells and the percentage of Ki67-positive cardiomyocytes. (E) Staining of Ki67 in WT mice or *beclin1*$^{+/-}$ mice heart post MI response to EMPA. Quantification was shown in (F). Data are shown as mean ±S.D. *P<0.05, **P<0.01, ***P<0.005.

Figure S12

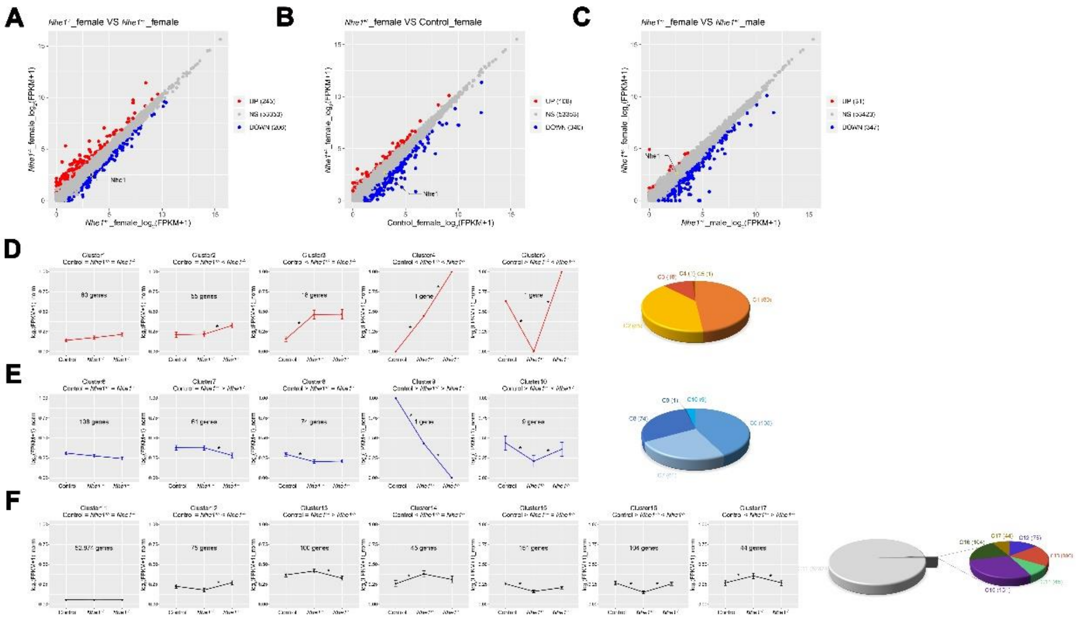

Transcriptome analyses of *Nhe1*$^{-/-}$, *Nhe1*$^{+/-}$ and WT mice heart tissue samples. (A-F) Identifying the expression patterns. GFOLD was used to call the differentially expressed genes (DEGs)

Figure S13

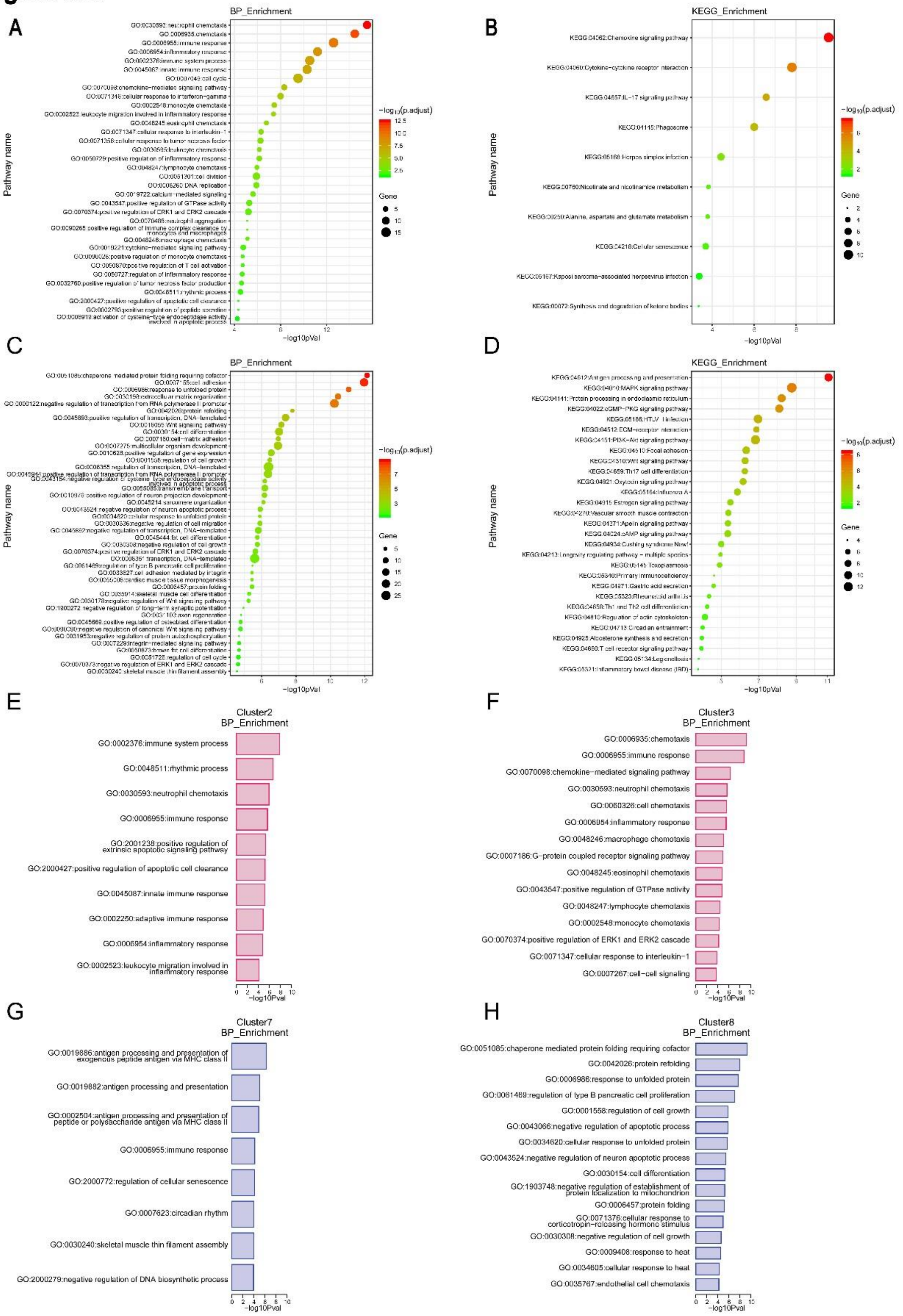

RNA-Seq analysis of gene expression. Gene up-regulated or down-regulated at *Nhe1* knockout and knockdown mice were subjected to functional enrichment analysis for KEGG pathways.

Table S3:
Sequences of qPCR primers

Gene	Forward primer (5'-3')	Reverse primer (5'-3')
SLC9A1	TCATCCACCTCGGATCTTCCC	TCCTGAGAACAGGTAGCAGTC
BECN1	ATGGAGGGGTCTAAGGCGTC	TCCTCTCCTGAGTTAGCCTCT
Atg4C	AGATGAAAGCAAGATGTTGCCT	CCCTGTAGGTCAGCCATATTCTA
Atg16L1	CAGAGCAGCTACTAAGCGACT	AAAAGGGGAGATTCGGACAGA
Map1lc3a	GACCGCTGTAAGGAGGTGC	CTTGACCAACTCGCTCATGTTA
Map1lc3b	TTATAGAGCGATACAAGGGGGAG	CGCCGTCTGATTATCTTGATGAG
Ulk1	AAGTTCGAGTTCTCTCGCAAG	CGATGTTTTCGTGCTTTAGTTCC
SQSTM1	AGGATGGGGACTTGGTTGC	TCACAGATCACATTGGGGTGC
Atg5	AGCCAGGTGATGATTCACGG	GGCTGGGGGACAATGCTAA
Atg7	GTTCGCCCCCTTTAATAGTGC	TGAACTCCAACGTCAAGCGG
Atg3	ACACGGTGAAGGGAAAGGC	TGGTGGACTAAGTGATCTCCAG
Lamp1	CAGCACTCTTTGAGGTGAAAAAC	ACGATCTGAGAACCATTCGCA
NFAT5	CAGCGCCCAATAGTTGGCA	TGCTGGTGAAAAATTGACTGGT
β-tubulin	GATCGGTGCTAAGTTCTGGGA	AGGGACATACTTGCCACCTGT

Table S4:
List of antibodies

Antibody	Catalogue Number	Supplier
LC3A/B Antibody	4108S	Cell Signaling Technology
P62 Antibody	5114	Cell Signaling Technology
NHE-1 Antibody	ab67314	Abcam
Anti-Ki67 antibody	ab16667	Abcam
Anti-Beclin1 Antibody	ab207612	Abcam
Alpha Actinin Antibody	ab9465	Abcam
GAPDH Antibody	2118S	Cell Signaling Technology
β-tubulin Antibody	2146	Cell Signaling Technology
Hsp90 Antibody	4874S	Cell Signaling Technology
